# Integrative machine learning analysis of bulk and single-nucleus RNA sequencing reveals key cell types and biomarkers driving MASH-associated fibrosis

**DOI:** 10.3389/fcell.2026.1961567

**Published:** 2026-10-29

**Authors:** Junjie Zhang, Junjie Wang, Yan Shen, Yirong Zhang, Zimeng Luo, Xinyao Kong, Wang Li, Juan Luo, Fei Xu, Shaogui Wan

**Affiliations:** 1 Key Laboratory of Prevention and Treatment of Cardiovascular and Cerebrovascular Diseases (Ministry of Education), Gannan Medical University, Ganzhou, China; 2 School of Basic Medicine, Gannan Medical University, Ganzhou, China; 3 Institute of Genomics and Precision Medicine, Gannan Medical University, Ganzhou, China; 4 School of Public Health and Health Management, Gannan Medical University, Ganzhou, China; 5 School of Medical and Information Engineering, Gannan Medical University, Ganzhou, China; 6 Department of Hepatology, The Affiliated Fifth People’s Hospital of Ganzhou, Gannan Medical University, Ganzhou, China; 7 School of Medical Technology, Gannan Medical University, Ganzhou, China

**Keywords:** cell state, intercellular communication, machine learning, MASH-associated fibrosis, snRNA-seq, TFs

## Abstract

**Background:**

Metabolic dysfunction-associated steatohepatitis (MASH) increases the risk of cirrhosis and liver cancer, with liver fibrosis being the primary determinant of adverse outcomes. However, the molecular mechanisms driving fibrosis progression in MASH remains incompletely understood.

**Methods:**

We integrated four bulk RNA-seq datasets into a merged cohort (MergeCohort) and analyzed one single-nucleus RNA-seq (snRNA-seq) dataset. Robust Rank Aggregation (RRA), WGCNA and machine learning were combined to identify core genes associated with MASH fibrosis. Additionally, we established MASH mice model induced by methionine-choline-deficient (MCD) diet, and further check the expression of hub genes through quantitative real-time PCR (qRT-PCR). Single-nucleus transcriptomic profiling of liver tissues from MASH patients across distinct fibrosis stages was investigated to delineate cellular heterogeneity, reconstruct intercellular communication networks, and characterize key transcription factors (TFs) as well as receptor-ligand interactions involved in MASH-associated fibrosis.

**Results:**

Based on the MergeCohort, we detected two key gene network modules associated with fibrosis progression in MASH through WGCNA. By integrating machine learning algorithms, we discovered seven core genes (CAPN6, FAP, KRT23, SOX4, STMN2, TAGLN and VWF) showing favorable discriminatory capability for fibrosis stage, achieving an area under the curve (AUC) of 0.81 (95% CI 0.69–0.93) in a distinct external cohort. Moreover, CAPN6, FAP and KRT23 showed directionally consistent upregulation in the liver tissues of MASH mouse model induced by MCD diet. Subsequently, applying cell-type deconvolution analysis, we assigned the predominant cell types (hepatic stellate cells (HSCs), cholangiocytes, hepatocytes and macrophages) to the key WGCNA modules. At the single-nucleus level, we identified FGF13^+^ cholangiocytes and SAAs^+^ hepatocytes subpopulations. Transcription factor regulon analysis identified key TFs (CREB5 and STAT3), which may play potential regulatory roles in FGF13^+^ cholangiocytes and SAAs^+^ hepatocytes. Cell-cell communication analysis further revealed crucial ligand-receptor pairs between HSCs and FGF13^+^ cholangiocytes (VEGFA_NRP1 and APP_CD74 axes), SAAs^+^ hepatocytes and HSCs (RSPO3_LGR4 and IGF1_IGF1R axes), SAAs^+^ hepatocytes and macrophages (TNFSF14_TNFRSF14 and SELE_CD44 axes) associated with the progression of liver fibrosis in MASH.

**Conclusion:**

This combined approach defines molecular signatures related to MASH fibrosis that may inform the development of novel therapeutic strategies and serve as a tissue signature associated with advanced fibrosis in MASH.

## 1 Introduction

MASH (previously named non-alcoholic steatohepatitis (NASH)) is a severe form of metabolic dysfunction-associated steatotic liver disease (MASLD; previously named non-alcoholic fatty liver disease (NAFLD)) characterized by hepatosteatosis, inflammation, and liver fibrosis, ultimately resulting in cirrhosis, liver transplantation, and hepatocellular carcinoma (HCC) (Friedman et al., 2018; Loomba et al., 2021). Among the diverse histological features of MASH, liver fibrosis according to the activation of collagen-producing hepatic stellate cells (HSCs) is the major predictor of mortality, establishing fibrosis as an important therapeutic target in MASH (Loomba et al., 2021; Tacke and Weiskirchen, 2021). However, current therapeutic options to ameliorate MASH-associated liver fibrosis are still lacking (Stephen A. et al., 2023; Harrison et al., 2024).

The pathogenesis of MASH fibrosis involves a complex crosstalk of multiple cell types within the liver, including hepatocytes, inflammatory cells, cholangiocytes, and HSCs (Loomba et al., 2021; Sharma et al., 2023; Lou et al., 2025). Single-cell transcriptomic technologies have advanced our understanding of liver cell types, states, and regulatory mechanisms (Aizarani et al., 2019; Ramachandran et al., 2019; Zhang et al., 2019). Recent advances in single-cell or single-nucleus RNA sequencing (sc/snRNA-seq) technologies have enabled researchers to uncover the cellular heterogeneity and intercellular crosstalk underlying MASH fibrogenesis (Wang et al., 2023; Gribben et al., 2024; Hong et al., 2025; Martin et al., 2025). RNA sequencing technology has been widely used in the study of MASH fibrosis, and its validity and reliability in the search for novel biomarkers for the diagnosis and treatment of MASH fibrosis have been demonstrated (Govaere et al., 2020; Kendall et al., 2023). However, a comprehensive, integrated analysis of transcriptional dynamics and cell-type-specific contributions across MASH fibrosis progression remains scarce. Therefore, such a study is critically needed to identify novel gene signatures and key cell subsets with unique disease-promoting or protective features for MASH fibrosis.

In this study, we integrated bulk and single-nucleus transcriptomic data to investigate the mechanisms underlying liver fibrosis in MASH. Our analysis included four human MASH RNA-Seq datasets and one snRNA-seq dataset obtained from previous studies. We applied differentially expressed gene (DEG), robust rank aggregation (RRA), coexpression network analysis, and machine learning methods to develop a 7-gene transcriptional signature to predict MASH fibrosis progression. Using cell-type deconvolution, we assigned the contributions of specific hepatic cell populations to the MASH fibrosis-associated modules. Utilizing marker genes of cellular states, we thoroughly characterized the functional landscape of crucial cell types linked to MASH fibrosis. We also identified critical cell subpopulations and associated upstream regulators, indicating that they may serve as potential biomarkers for MASH fibrosis. In addition, we elucidated the specific cell-to-cell interactions among cholangiocytes, hepatocytes, HSCs, and macrophages in MASH fibrosis. These findings offer valuable insights into the pathogenesis of MASH fibrosis at the molecular and cellular levels and highlight potential therapeutic targets.

## 2 Materials and methods

### 2.1 Manually collection of MASH transcriptomes

Bulk and single-nucleus transcriptomic data of liver tissues from MASH were obtained from the Gene Expression Omnibus (GEO, https://www.ncbi.nlm.nih.gov/geo/) database. Specifically, our study included four human RNA-seq datasets (GSE130970, GSE135251, GSE162694, GSE225740 and GSE174478), and one human snRNA-seq dataset (GSE202379). To further validate the gene expression of key biomarkers based on fibrosis stage, we utilized the SteatoSITE (https://shiny.igc.ed.ac.uk/SteatoSITE_gene_explorer/), which is an open resource comprising transcriptomic data from 940 histologically confirmed patients encompassing MASLD/MASH spectrum (Kendall et al., 2023). Additionally, to estimate the gene expression of key genes in mouse MASH models, we also retrieved 7 mouse transcriptomic datasets (GSE205846, GSE137449, GSE114261, GSE162863, GSE233767, GSE162869 and GSE119340) from the GEO database. Detailed information related to these datasets is provided in Supplementary Table S1.

### 2.2 Differentially expressed gene (DEG) analysis and robust rank aggregation (RRA) analysis

MASH was classified according to fibrosis stage into MASH with advanced fibrosis (MASH F3-4) and MASH with mild fibrosis (MASH F0-2). DEG analysis for each human RNA-seq dataset was conducted using the R package DESeq2 (v.1.46.0) (Love et al., 2014). *P* values were adjusted using the Benjamini–Hochberg method. Genes were considered significant DEGs if they presented an adjusted *P* value less than 0.05 and an absolute value of log_2_fold change greater than log_2_ (1.5). The Robust Rank Aggregation (RRA) analysis was subsequently applied to integrate the DEG analysis results from multiple datasets, implemented using the R package RobustRankAggreg (v.1.2.1) (Kolde et al., 2012). After RRA, the average log_2_FC across datasets was calculated for the significant genes identified by the integration and subjected to a secondary filtering step. We used Bonferroni multiple testing correction to adjust raw *P* values. Genes with an adjusted *P* < 0.05 and an absolute log_2_FC > 0.30 were defined as DEGs, consistent with previous studies (He et al., 2023).

### 2.3 Batch effect removal and mergecohort construction

After removing low-expression genes and outlying samples, we employed the ComBat method within the R package sva (v.3.54.0) to mitigate batch effects resulting from technical variations between individual human RNA-seq and retain biological variation among samples (Leek et al., 2012). The model was written as ComBat(dat = expr_matrix, batch = dataset, mod = model.matrix (∼fibrosis_stage), par.prior = TRUE). Here, batch corresponds to the dataset/cohort variable, and mod explicitly includes fibrosis stage as a biological covariate of interest. This process combined multiple datasets into a single expression matrix, referred to as MergeCohort. Collectively, the MergeCohort contained 331 individuals, including 219 with MASH with mild fibrosis and 112 with advanced fibrosis. Principal component analysis (PCA) was performed using the FactoMineR package (v.2.12), and visualization was performed using the fviz_pca_ind function from the factoextra package (v.1.0.7). Based on the MergeCohort expression matrix, DEGs between two groups of samples were identified using the Wilcoxon rank-sum test. Significance was defined as a Benjamini–Hochberg-adjusted *P* value under 0.05 and an absolute log_2_FC exceeding 0.30.

### 2.4 Weighted gene coexpression network analysis (WGCNA)

WGCNA analysis was performed using the WGCNA R package (v.1.73) (Langfelder and Horvath, 2008). 5,000 genes exhibiting the highest variability across all patients with MASH (*n* = 331) based on median absolute deviation were selected from the batch-corrected expression matrix in the MergeCohort, which were included for subsequent coexpression network analysis. A soft thresholding power of 10 was chosen to construct the adjacency matrix, satisfying the criterion of an approximate scale-free topology (fit index R^2^ > 0.85). For network construction and module identification, the blockwiseModules function was applied with the following parameters: DeepSplit = 4, corType = “pearson”, networkType = “signed hybrid”, mergeCutHeight = 0.25, and minModuleSize = 30. Finally, Pearson correlation analysis was used to evaluate the associations between the identified module eigengenes and stage along with the MASH fibrosis score.

### 2.5 Gene set enrichment analysis (GSEA)

Gene set enrichment analysis (GSEA) was conducted using the R package fgsea (v.1.32.4) (Korotkevich et al., 2021). The MASH fibrosis signature was derived from the differential transcriptome between patients with advanced MASH and those with mild MASH in the MergeCohort. Genes were ranked according to the *P* value obtained from the Wilcoxon rank-sum test to compare advanced versus mild fibrosis. In accordance with the GSEA methodology, no pre-filtering of genes, such as expression level, variance, or statistical significance, was applied prior to enrichment analysis. The MASH fibrosis signature was subsequently tested for enrichment in the coexpression modules identified by WGCNA.

### 2.6 Functional enrichment analysis

In order to investigate the possible functions of modular genes and DEGs, enrichment was tested using the implementation of the Enrichr (Kuleshov et al., 2016) workflow in Python package gseapy (https://github.com/zqfang/gseapy;v.1.1.2) (Fang et al., 2023) with “GO_Biological_Process_2021” “KEGG_2021_Human” and “MSigDB_Hallmark_2020” as query databases. The significance threshold was set at a *P* value of 0.05.

### 2.7 Gene set variation analysis (GSVA)

To evaluate changes in molecular features within individual samples, GSVA enrichment scores were computed for gene sets comprising established diagnostic biomarkers associated with MASH fibrosis stage by using the R package GSVA (v.2.0.7) (Hanzelmann et al., 2013). The resulting GSVA enrichment scores provided an indication of the extent to which these molecular features were increased or decreased in MASH liver tissues.

### 2.8 Generation of potential biomarkers based on machine learning algorithms

By integrating the results from the RRA, DEGs, and WGCNA analyses, we identified overlapping genes associated with MASH fibrosis. To screen for biomarkers among these overlapping genes, we employed multiple machine learning algorithms including Least Absolute Shrinkage and Selection Operator (LASSO) regression, Random Forest (RF), and Support Vector Machine-Recursive Feature Elimination (SVM-RFE). LASSO regression was performed using the glmnet R package (v.4.1-10) with 10-fold cross validation (Friedman et al., 2010). The RF algorithm was implemented using the randomForest R package (v.4.7-1.2) and “ntree” set at 500 (Rigatti, 2017). SVM-RFE was conducted using the e1071 R package (v.1.7-16) with 10-fold cross validation (Sanz et al., 2018; Abinash and Vasudevan, 2022). The optimal diagnostic biomarkers for MASH fibrosis were ultimately determined based on the intersection of features selected by these three machine learning methods.

### 2.9 Validation and predictive evaluation of the potential biomarkers

To evaluate the predictive ability of potential biomarkers in discriminating advanced fibrosis from mild fibrosis in MASH, we first conducted GSVA analysis in a genuinely independent cohort (GSE174478) (Kawamura et al., 2022). Subsequently, the R package pROC (v.1.19.0.1) was used to create receiver operating characteristic (ROC) curves for that dataset (Robin et al., 2011), and the area under the curve (AUC) values were computed to quantify the classification accuracy. Furthermore, we verified the expression patterns of key biomarkers during the fibrosis stage based on the SteatoSITE database.

### 2.10 snRNA-seq data processing and analysis

For the snRNA-seq data analysis, the raw count matrices were obtained from the GEO database under accession number GSE202379, comprising transcriptomic profiles of 31 liver biopsies from MASH patients (Gribben et al., 2024). Seurat (v.4.4.0) package was used for downstream analysis (Hao et al., 2021). For quality control, we excluded nuclei with fewer than 800 features, mitochondrial content exceeding 10%, and ribosomal content above 10%. Only nuclei with a total feature count greater than 1,000 were retained. To further eliminate potential doublets, nuclei with >50,000 total counts were discarded. Additionally, potential doublets were detected using the scDblFinder (v. 1.20.2) package (Germain et al., 2022), and contamination from ambient RNA was identified using DecontX (v.1.4.1) (Yang et al., 2020). Our analysis yielded 50,605 high-quality nuclei from 31 MASHpatients with fibrosis. The raw counts were normalized using Seurat’s SCTransform function. From this, the principal components (PCs) were computed using the top 3,000 variable features with the RunPCA function. The top 30 PCs were selected to construct a shared nearest-neighbor graph using the FindNeighbors function. Clustering was then performed using the FindClusters function with the Louvain algorithm. We applied Uniform Manifold Approximation and Projection (UMAP) for dimensionality reduction using the RunUMAP function. The broad cell type classes were annotated based on the expression of canonical marker genes (Supplementary Table S11). To account for batch effects and inter-sample heterogeneity, Harmony was applied for data integration with the RunHarmony function (Tran et al., 2020). Individual clusters corresponding to hepatocytes, cholangiocytes, and stellate were subsets in separate objects for re-clustering. Each of these subtypes was then reanalyzed independently, following the processes described above. To compare pseudobulk gene expression across different fibrosis stages in cell types of interest, we performed sample-wise pseudobulk aggregation of raw counts using the AggregateExpression function from Seurat. Normalization and differential expression analysis were performed with DESeq2 (v.1.46.0), considering genes with adjusted *P* value <0.05 and |log_2_fold change| > log_2_ (1.5) as differentially expressed. To detect general cluster markers, FindMarkers was used to compare cluster of interest against all others using the default Wilcoxon rank-sum test. *P* values were adjusted using the Bonferroni correction. Genes with an adjusted *P* value less than 0.05 and an absolute log_2_FC greater than 0.25 were considered significant DEGs. Cell-type abundance differences between advanced and mild fibrosis MASH patients were initially assessed using the Wilcoxon rank-sum test, followed by validation with scCODA (v.0.1.9) (Büttner et al., 2021), a Bayesian framework for compositional data analysis that accommodates variable cell numbers across patients, with automatic selection of the reference cell type. A cell type was deemed differentially abundant only if it reached significance in both analyses (Wilcoxon *P* < 0.05 and scCODA 95% highest density interval excluding zero).

### 2.11 Cell type deconvolution of bulk RNA-seq data

To estimate the proportions of major cell types in bulk RNA-seq data from the MergeCohort, we employed CIBERSORTx (Newman et al., 2019). Specifically, annotated single-cell reference matrix files were created from each cell-type cluster in the snRNA-Seq dataset with Seurat’s GetAssayData function. The single-cell expression matrix file was then used to generate the signature matrix using the Create Signature Matrix module on the CIBERSORTx server (https://cibersortx.stanford.edu/) with default settings and a minimal expression set to 0. The batch-corrected bulk RNA-seq gene expression matrix was also submitted to the CIBERSORTx server, and the Impute Cell Fractions module was applied to infer the cell type abundance in individual samples from the MergeCohort. S-mode batch correction and disabled quantile normalization options were performed for the signature matrix and bulk RNA-seq, respectively. However, a potential limitation of our deconvolution approach is the use of a single-nucleus RNA-seq reference, which captures only nuclear transcripts and may not fully recapitulate the cytoplasmic transcriptome present in bulk RNA-seq data.

The permutations for significance analysis were set at 500. To explore the cell-type attribution for each module identified by WGCNA, we computed Pearson correlations between the deconvoluted cell fractions calculated by CIBERSORTx and module eigengenes across samples.

### 2.12 Cell-cell interaction analysis

CellPhoneDB (v.2.1.7) was used to infer cell-cell communications within the snRNA-seq dataset of MASH with fibrosis (Efremova et al., 2020). CellPhoneDB is a curated database of ligands, receptors, and their interactions, integrated with a statistic framework for inferring significant cell-cell interaction networks from sc/snRNA-seq data. After identifying different cell types, we performed cell-cell interaction analysis with a local implementation of CellPhoneDB, followed the recommended protocol for data preparation (Vento-Tormo et al., 2018; Efremova et al., 2020). In brief, we extracted the SCTransform-normalized expression matrix and metadata from the corresponding Seurat object. CellPhoneDB analysis was performed using ‘cellphonedb method statistical analysis’ command with default parameters. For each analysis run, a summary output file was generated, which compiled the interactions for each cell pair (*P* < 0.05) and adjusted *P* values with Benjamini–Hochberg correction for multiple testing.

### 2.13 Transcription factor regulon analysis with SCENIC

The R package SCENIC (v.1.3.1) was used to identify the regulatory transcription factor genes (also called regulons) of cell subpopulations (Aibar et al., 2017). As the first step of the SCENIC workflow was identifying the potential gene regulatory network (GRN) using runGenie3 () and runSCENIC_1_coexNetwork2modules () functions, followed by the selection of candidate regulons via runSCENIC_2_createRegulons (), which incorporated hg38__refseq-r80__500bp_up_and_100bp_down_tss.mc9nr.feather motif dataset retrieved from the cisTarget repository (https://resources.aertslab.org/cistarget/). Finally, regulon activity was scored across individual cells using runSCENIC_3_scoreCells (), and regulon specificity scores (RSS) for each cell subset were computed using the calcRSS() function.

### 2.14 Animals and treatment

Male C57BL/6 (8 weeks old) mice were purchased from Jiangsu Huachuang Sino Pharmaceutical Technology Co., Ltd. (Taizhou, China) and maintained under specific pathogen-free (SPF) conditions. The animal experiments were conducted in accordance with Guide for the Care and Use of Laboratory Animals, published by the US National Institute of Health and supervised by the Institutional Animal Care and Use Committee of Gannan Medical University (Approval No. 202219). The experiment was divided into 3 groups (CON, MCD 4W, MCD 6W), with 6 mice in each group. To establish MASH, mice were fed with methionine and choline-deficient (MCD) diet (MD12052, Medicience, Yangzhou, China) for 4 or 6 weeks. At the experimental endpoint, mice were euthanized under isoflurane (2%-3%) anesthesia prior to cervical dislocation in accordance with the approved institutional animal care protocol. The liver tissues were immediately harvested for further analysis.

### 2.15 Histopathological staining

Liver tissues were excised and immediately fixed in 10% neutral-buffered formalin, then embedded in paraffin and cut into 5 μm sections. Deparaffinized and rehydrated liver sections were stained with hematoxylin and eosin (H&E) or Sirius Red following standard protocols. For Oil Red O staining, frozen liver sections were cut into 10 μm, fixed in acetone for 10 min, and washed with phosphate-buffered saline (PBS) for 3 min prior to staining.

### 2.16 Quantitative real-time polymerase chain reaction (qRT-PCR)

RNA isolation and qRT-PCR were performed according to the manufacturer’s instructions. Briefly, total RNA of livers for normal and MCD diet mice was extracted with TRIzol reagent (CW3166M, CWBIO, China). RNA was then reverse-transcribed into cDNA using NovoScriptPlus All-in-one first Strand cDNASynthesis SuperMix (gDNA Purge) (E047, Novoprotein, China). qRT-PCR was performed using the NovoStart® Universal Fast SYBR qPCR SuperMix (E401, Novoprotein, China) according to the manufacturer’s instruction. The relative expression level of key gene was normalized to murine glyceraldehyde 3-phosphate dehydrogenase (GAPDH) according to the 2^−ΔΔ*Ct*^ method. The primers were detailed in Supplementary Table S10.

### 2.17 Statistical analyses

All statistical analyses throughout the study were performed using R software (v.4.4.1). Comparison of GSVA scores between the two groups was performed using an unpaired Student’s t*-*test. The Pearson’s correlation coefficient was used to characterize the correlation. Statistical significance was set at *P* < 0.05.

### 2.18 Visualization

We used the R package ggplot2 (v.3.5.2) and Seurat to perform most visualizations, and the pheatmap package (v.1.0.13) was used to create heatmaps.

## 3 Results

### 3.1 Transcriptomic changes during fibrosis progression in MASH

A total of four liver fibrosis-related RNA-seq datasets (GSE130970, GSE135251, GSE162694, and GSE225740) were included as discovery cohort in this study, comprising 254 MASH patients with mild fibrosis (F0–2) and 122 patients with advanced fibrosis (F3–4) (Supplementary Table S1). Specifically, GSE130970 cohort consists of 46 (31 mild fibrosis and 15 advanced fibrosis) patients, derived from 14 (13 mild fibrosis and 1 advanced fibrosis) males and 32 (18 mild fibrosis and 14 advanced fibrosis) females. GSE135251 cohort comprises 155 (87 mild fibrosis and 68 advanced fibrosis) patients, including 87 (49 mild fibrosis and 38 advanced fibrosis) males and 68 (38 mild fibrosis and 30 advanced fibrosis) females. GSE162694 cohort includes 112 (92 mild fibrosis and 20 advanced fibrosis) patients, comprising 37 (28 mild fibrosis and 9 advanced fibrosis) males and 75 (64 mild fibrosis and 11 advanced fibrosis) females. GSE225740 cohort comprises 63 (44 mild fibrosis and 19 advanced fibrosis) patients, consisting of 25 (21 mild fibrosis and 4 advanced fibrosis) males and 38 (23 mild fibrosis and 15 advanced fibrosis) females. After data filtering and batch effect correction, we created a batch-corrected bulk RNA-seq expression matrix (MergeCohort) covering 219 samples from MASH patients with mild fibrosis and 112 samples from individuals with advanced fibrosis from the four independent datasets. PCA plots with or without elimination of batch effects are shown in Supplementary Figure S1. Clean separation of samples in the source datasets was observed prior to batch correction, suggesting the presence of batch effects (Supplementary Figure S1A). However, after applying ComBat for batch correction, the samples were uniformly distributed (Supplementary Figure S1B), indicating successful removal of these technically driven variations. The PCA of the MergeCohort gene expression matrix distinguished samples with advanced fibrosis from those with mild fibrosis (Supplementary Figure S1C). DEG analysis identified 488 upregulated and 125 downregulated genes in advanced fibrosis compared to mild fibrosis (Supplementary Figure S1D; Supplementary Table S2). To confirm that batch correction did not inflate DEG significance, we compared MergeCohort DEGs with per-dataset DESeq2 results. The two approaches yielded significantly overlapping DEG sets (Supplementary Figure S2) and concordant log_2_fold changes across four datasets (Supplementary Figure S3). GO enrichment analysis revealed that the upregulated genes were associated with various ECM-related processes, including extracellular structure organization, extracellular matrix organization, supramolecular fiber organization, and collagen fibril organization (Supplementary Figure S1E; Supplementary Table S3). The downregulated genes were significantly enriched for GO terms related to lipid and amino acid metabolism (Supplementary Figure S1F; Supplementary Table S4).

### 3.2 Coexpression network analysis reveals modules related to MASH fibrosis stage

To identify the key gene modules that were contributing to the progression of liver fibrosis in MASH, we performed coexpression network analysis using WGCNA in the MergeCohort dataset. During the WGCNA procedure, a soft threshold power β of 10 (scale-free R^2^ = 0.87) was selected as the optimal power value for constructing coexpression networks (Supplementary Figure S4). WGCNA identified 10 coexpression gene modules, each denoted by a unique color (Figure 1A). Each module is named with the numeric module label, module size, and top enrichment term (e.g., “M3 (347): Extracellular matrix organization”) (Figure 1B; Supplementary Table S5). The expression profile of genes within each module was summarized by the module eigengene (the first principal component), which captured the majority of variance in module gene expression across samples. Module sizes varied between 39 and 407 genes, as shown in Figure 1B. Next, we evaluated the correlations between module eigengenes and the MASH fibrosis stages or scores. Notably, we found that four modules (M1 (66): Immune response, M3 (347): Extracellular matrix organization, M6 (51): Protein targeting to ER and M8 (74): Epithelial cell morphogenesis) were significantly associated with MASH score and fibrosis grade (Figure 1B). Furthermore, a relatively strong correlation was observed between gene significance (GS) and module membership (MM) specifically in the M3 and M8 modules (Figures 1C,D). Gene Set Enrichment Analysis (GSEA) further revealed that genes from the M3 and M8 modules were most highly enriched for genes upregulated in MASH with advanced fibrosis (Figure 1E). Finally, functional annotation via GO enrichment analysis showed that genes in the M3 module were primarily involved in biological processes, such as ECM organization and cell migration, while genes in the M8 module were mainly associated with epithelial morphogenesis, epithelial development, and the regulation of epithelial cell migration (Figure 1F; Supplementary Table S6). Together, these findings suggest that the M3 and M8 modules are not only associated with MASH fibrosis severity but are also enriched for genes whose dysregulation might associated with the progression of MASH fibrosis.

**FIGURE 1:**
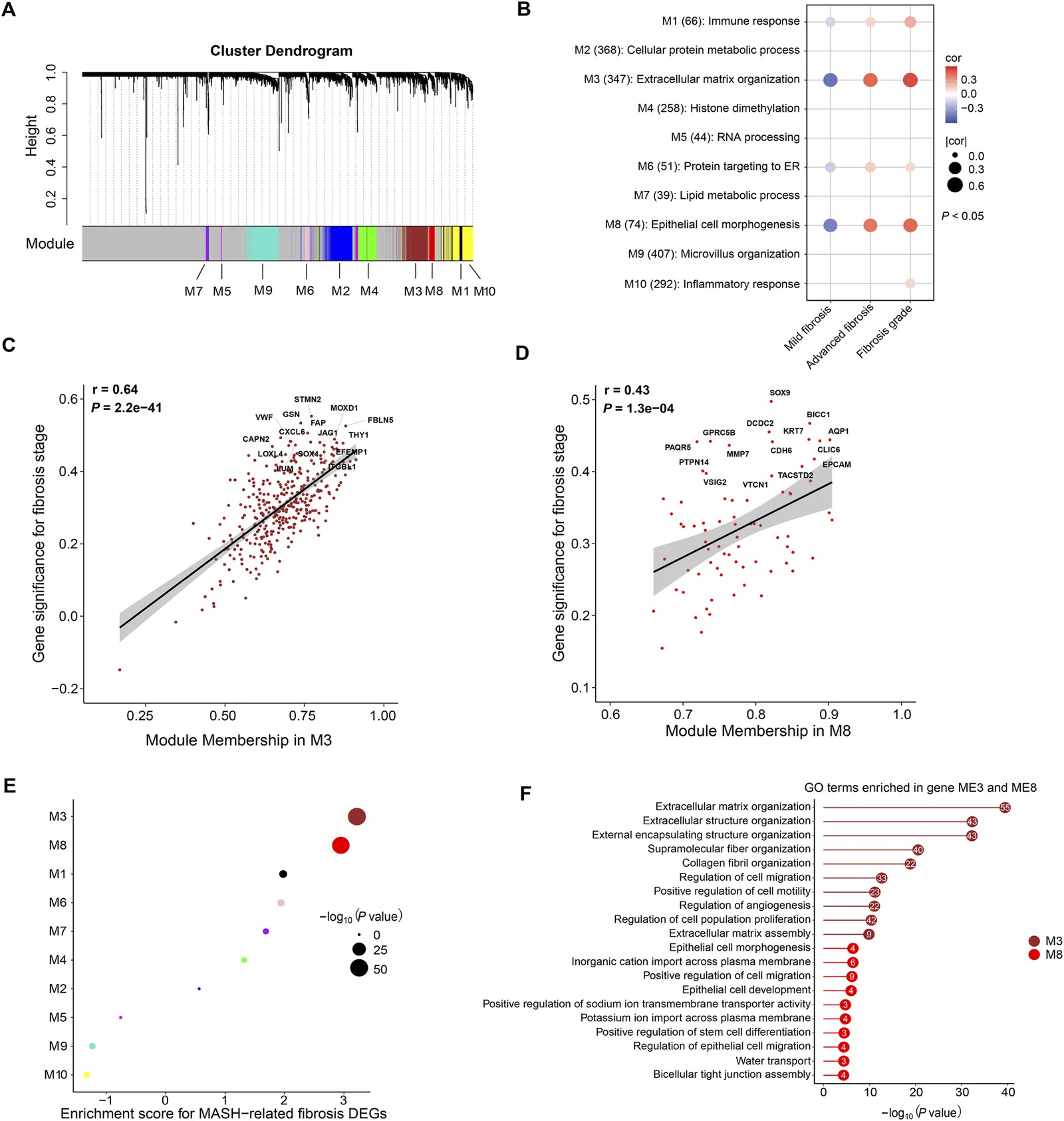
Coexpression network analysis reveals modules associated with liver fibrosis stage in MASH. **(A)** Gene clustering dendrogram determined by weighted gene coexpression network analysis (WGCNA) with color module assignments shown at the bottom. **(B)** Heatmap displaying significant (*P* < 0.05) Pearson correlations between module eigengenes with fibrosis severity, red and blue represent positive and negative correlation, respectively. Each row corresponds to a module. Row labels display the numeric module label, module size, and the most enriched term. Module eigengenes were directionally aligned to mean expression of module genes prior to correlation analysis. Dot size corresponds to correlation strength. **(C, D)** Scatter plots depicting the correlation between gene significance for fibrosis stage and module membership of genes in the M3 and M8 modules, respectively. The top 15 hub genes are labelled. **(E)** Dot plot demonstrating the normalized enrichment score of modules for the MASH differential transcriptome as determined by Gene set enrichment analysis (GSEA). Dot size indicates statistical significance (-log_10_ (*P* value)). **(F)** Lollipop plots showing the top ten GO terms of biological processes enriched by genes in the M3 and M8 modules.

### 3.3 Transcriptomic risk prediction of fibrosis stage in MASH

RRA analysis revealed 88 upregulated and 14 downregulated DEGs in MASH patients with advanced fibrosis (Supplementary Table S7). An overlap of 74 genes was observed between the RRA-identified DEGs, DEGs from the MergeCohort, and genes within the crucial WGCNA modules (Figure 2A; Supplementary Table S8). KEGG enrichment analysis revealed that the overlapping genes were strongly associated with chemokine signaling, cytokine-cytokine receptor interaction, IL-17 signaling, TNF signaling, and ECM-related pathways (Figure 2B; Supplementary Table S9). To refine the candidate gene set, we applied three machine-learning methods (LASSO, SVM-RFE, and RF) for feature selection. The LASSO algorithm selected 18 genes (Figures 2C,D), the RF analysis yielded 27 genes (Figures 2E,F) and SVM-RFE highlighted 31 genes (Figures 2G,H). Intersection of the results via Venn diagram identified seven hub genes (CAPN6, FAP, KRT23, SOX4, STMN2, TAGLN, and VWF) for subsequent analysis (Figure 2I). We then conducted gene set variation analysis (GSVA) based on the expression of the 74-gene and 7-gene signature in an independent external cohort (GSE174478). Because the GSE174478 cohort lacks information on steatosis, ballooning, and lobular inflammation, MASLD patients could only be provisionally classified as MASH based on a non-alcoholic fatty liver disease activity score (NAS) ≥ 5. Using this criterion, we selected 56 patients with MASH, including 35 with mild fibrosis and 21 with advanced fibrosis, comprising 27 males (18 mild fibrosis and 9 advanced fibrosis) and 29 females (17 mild fibrosis and 12 advanced fibrosis). The 7-gene signature showed significantly elevated unweighted gene-set enrichment scores in advanced fibrosis (*P* = 6.8e-05) and achieved an AUC of 0.81 (95% CI 0.69 to 0.93) in this independent cohort (Figure 2K), which was comparable to the AUC of 0.84 (95% CI 0.73 to 0.94) of the 74-gene panel in the same cohort (Figure 2J), indicating that reducing the panel from 74 to 7 genes largely preserves its discriminative performance. To further confirm the expression of the seven key genes in a separate cohort, we used SteatoSITE (https://shiny.igc.ed.ac.uk/SteatoSITE_gene_explorer/). In this dataset, we observed that these genes exhibited increased expression during fibrosis (Figure 2L). Moreover, to assess whether sex confounded our DEG results for this 7-gene signature, we included sex as a covariate in the design formula (∼sex + condition) and compared the log_2_fold change and adjusted *P* value before and after sex adjustment in each discovery cohort. After sex adjustment, KRT23 and STMN2 lost significance in GSE130970 cohort, while STMN2 and TAGLN were no longer significant in GSE225740 cohort (Supplementary Figure S5). This attenuation may partly attributable to the relatively small number of male patients in MASH with advanced fibrosis group in both GSE130970 (7% male in advanced fibrosis versus 42% male in mild fibrosis) and GSE225740 (21% male in advanced fibrosis versus 48% male in mild fibrosis) cohorts.

**FIGURE 2:**
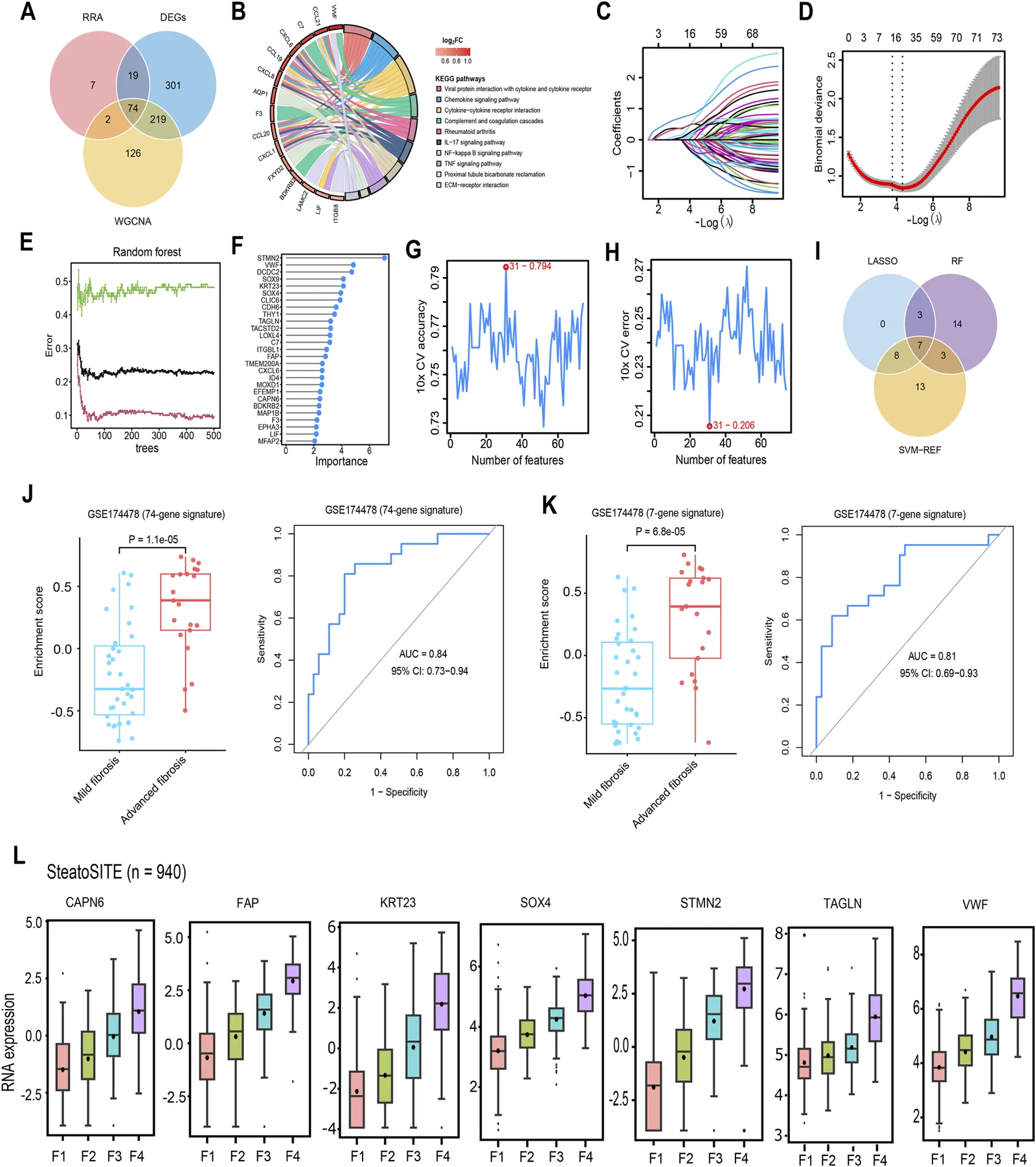
Identification and validation of hub genes associated with liver fibrosis progression in MASH. **(A)** Venn diagram showing the number of genes overlapped between RRA-identified DEGs, DEGs observed in MergeCohort and genes in the crucial WGCNA modules. **(B)** Chord diagram demonstrating the KEGG pathways enriched by overlapping genes. Each line represents a pathway, and the box color represents log_2_FC value of overlapping genes in MergeCohort. **(C, D)** Least absolute shrinkage and selection operator (LASSO) regression for hub gene screening. **(C)** LASSO coefficient spectrum for 18 genes, generating a logarithmic coefficient map. **(D)** Optimal lambda selection by repeated tenfold cross-validation, guiding feature selection. **(E, F)** Random forest (RF) for candidate hub gene identification. **(E)** Decision tree count impacts error rates. Green, red, and black denote MASH with advanced fibrosis, MASH with mild fibrosis, and all samples respectively. **(F)** Gini importance plot with mean decrease Gini on the x-axis and hub genes on the y-axis. **(G, H)** Finalizing 31 characteristic hub genes using Support Vector Machine-Recursive Feature Elimination (SVM-RFE). **(I)** Venn diagram showing the overlapping hub genes identified by the three machine learning methods. **(J, K)** Comparison of the unweighted gene-set enrichment score and AUC value achieved using the 7-gene signature with that of the original 74-gene signature in a genuinely independent cohort (GSE174478). *P* values are determined by unpaired student’s t*-*test. **(L)** Boxplots displaying the expression patterns of the seven hub genes during MASLD fibrosis stages based on SteatoSITE (https://shiny.igc.ed.ac.uk/SteatoSITE_gene_explorer/), an open resource encompassing histologically characterized data from 940 MASLD.

### 3.4 Cross-species expression consistency check of hub genes in a MASH mouse model

Furthermore, we performed a cross-species expression consistency check of the seven-gene signature in mice fed a MCD diet for 4 or 6 weeks. Mice fed a MCD for 4 or 6 weeks exhibited lower body weight and liver weight than the control mice, but liver/body weight ratio was not affected by MCD diet (Figure 3A). Histology analysis of liver tissue from mice using H&E and Oil Red O revealed that MCD diet induced obviously steatosis compared with control group, and severe lipid accumulation was observed in mouse fed with MCD diet for 6 weeks compared to mice fed for only 4 weeks (Figure 3B). Besides, Sirius red staining indicated that hepatic fibrosis were significantly aggravated in MCD diet group (Figure 3B). The mRNA expression of Capn6, Fap and Krt23, particularly Capn6, was significantly increased in the livers of mice fed with MCD diet for 6 weeks compared to mice fed a normal diet or MCD diet for 4 weeks (Figure 3C). In contrast, no significant differences were observed in Sox4, Stmn2, Tagln and Vwf at the transcript level in this mouse model (Figure 3C). Notably, after applying Bonferroni *post hoc* correction, Sox4 (*P* = 0.041 versus adjusted *P* = 0.12) no longer reached statistical significance when comparing mice fed with MCD diet for 6 weeks compared to mice fed MCD diet for 4 weeks, and Vwf (*P* = 0.042 versus adjusted *P* = 0.13) lost significance when comparing mice fed with MCD diet for 6 weeks compared to mice fed normal diet. However, it is important to acknowledge certain limitations in using the MCD model, which captures diet-driven steatohepatitis rather than the full metabolic and staging context of human MASH (Rinella and Green, 2004). Overall, the roles of these core genes in MASH fibrosis warrant further investigation.

**FIGURE 3:**
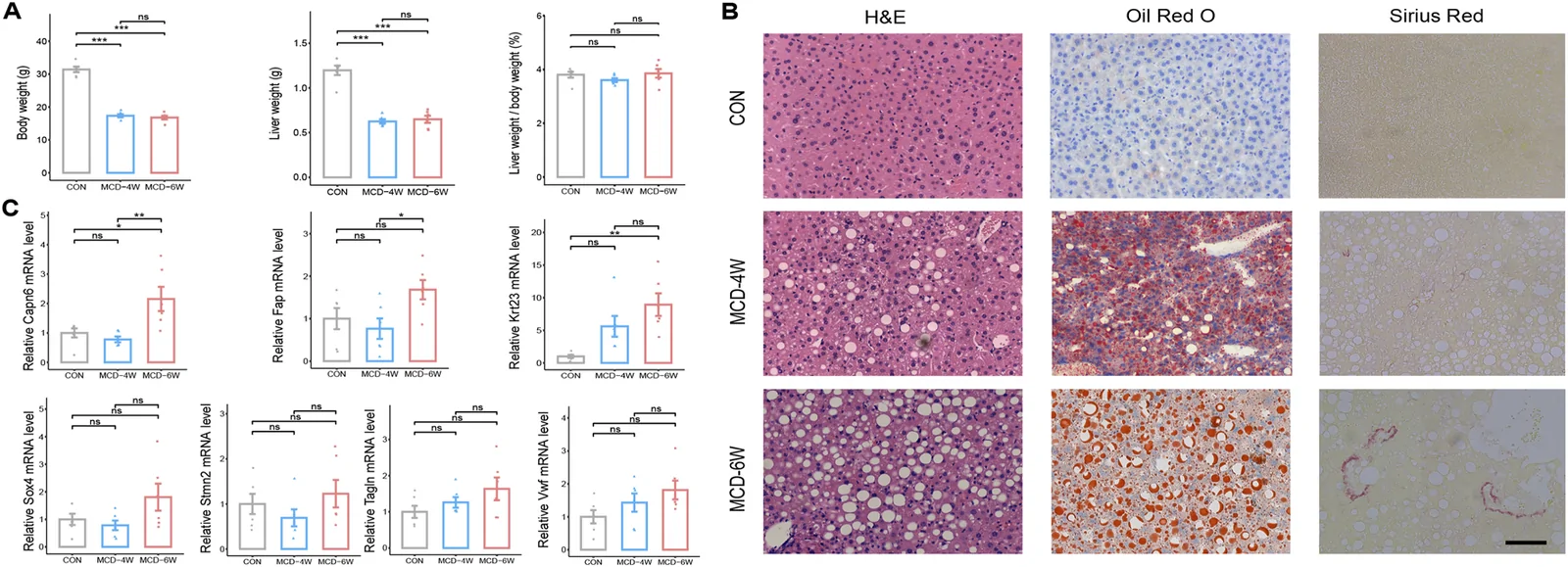
Animal experiment. **(A)** Body weights, liver weights and liver/body weight ratio of C57BL/6 mice fed with a normal chow diet or MCD diet for 4 or 6 weeks, starting at 8 weeks of age. **(B)** Liver histology analysis, including H&E, oil red O and sirius staining. Scale bar: 50 μm. **(C)** Relative mRNA levels of seven key genes in the livers of three groups of mice (n = 6 per group). ^*^ *P* < 0.05, ^**^ *P* < 0.01 and ^***^ *P* < 0.001 by one-way ANOVA with bonferroni *post hoc* analysis. ns, not significant.

### 3.5 Cell type deconvolution of modules associated with MASH fibrosis stage

To explore the potential cell types associated with WGCNA modules, we conducted deconvolution analysis using a transcriptomic reference derived from a publicly available human liver snRNA-seq dataset (GSE202379) (Gribben et al., 2024). This cohort comprised liver biopsies from 31 (16 mild fibrosis and 15 advanced fibrosis) patients, derived from 18 (8 mild fibrosis and 10 advanced fibrosis) males and 13 (8 mild fibrosis and 5 advanced fibrosis) females. After processing, we obtained 24,366 cells from 15 patients with advanced fibrosis and 26,239 cells from 16 patients with mild fibrosis. From gene expression of canonical markers (Supplementary Table S11), we identified hepatocytes, cholangiocytes, endothelial cells, lymphocytes, macrophages, hepatic stellate cells (HSCs), neutrophils, plasma cells, and B cells (Figure 4A). Comparative analysis revealed differences in cellular composition between the mild and advanced fibrosis groups (Figures 4C–E), and expression patterns of cell type-specific marker genes were visualized across these populations (Figure 4B). The proportional abundance of each cell type among MASH patients was further compared (Figure 4D). Using a reference signature matrix from this snRNA-seq atlas, we deconvoluted bulk transcriptome from the MergeCohort through CIBERSORTx (Newman et al., 2019). To integrate cellular composition with coexpression modules, correlation analysis was conducted between the inferred cell-type fractions and module eigengenes across all samples. Consistent with the clinical fibrosis stage, significant positive correlations were observed between the M3 (347): Extracellular matrix organization, M8 (74): Epithelial cell morphogenesis modules and HSCs, together with the association of the M3 (347): Extracellular matrix organization, M8 (74): Epithelial cell morphogenesis modules with cholangiocytes (Figures 4F,G). Additionally, the M1 (66): Immune response module correlated most strongly with macrophages, whereas the M6 (51): Protein targeting to ER module was predominantly associated with hepatocytes (Figures 4F,G).

**FIGURE 4:**
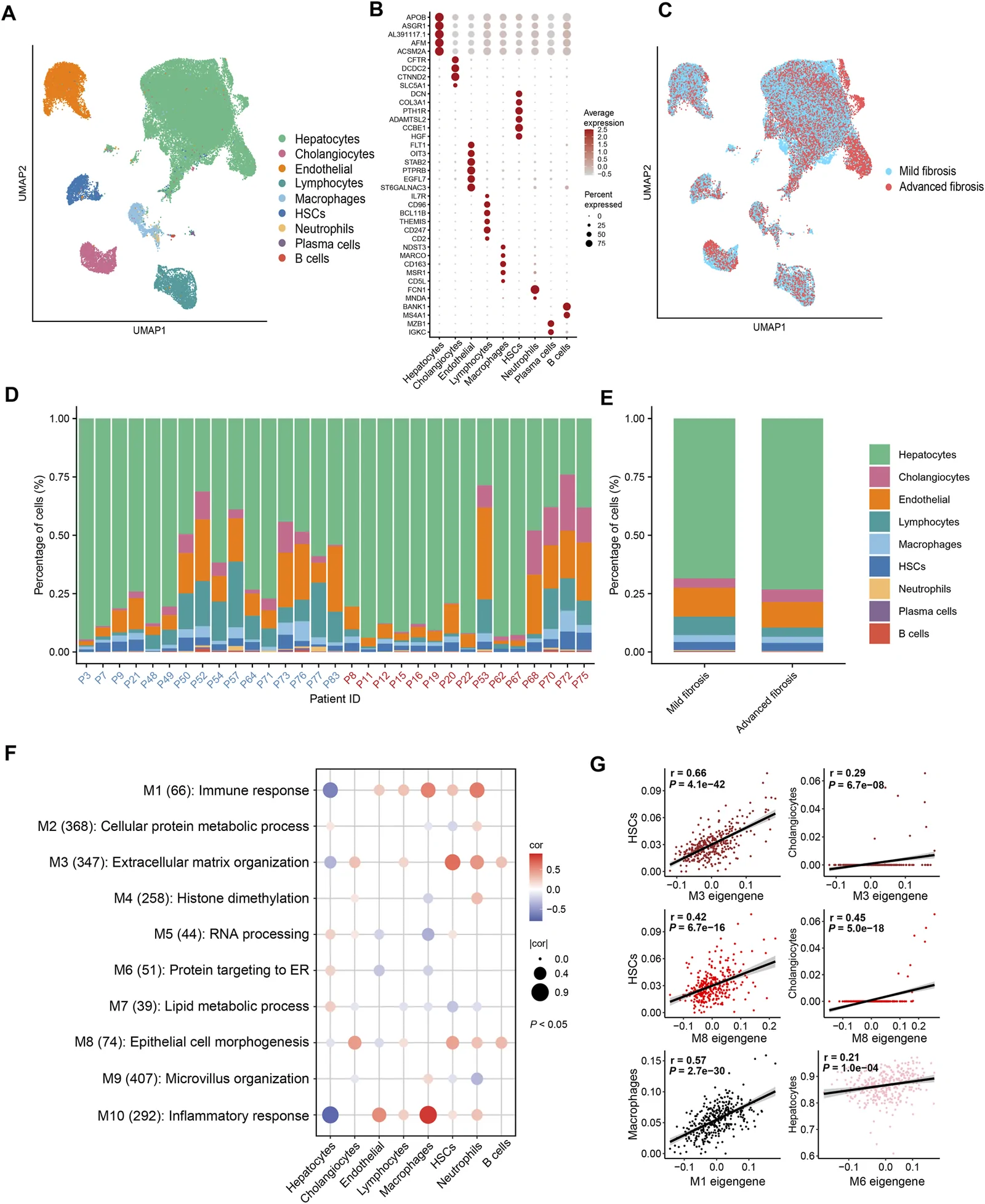
snRNA-seq analysis on the MASH livers with fibrosis and relative contributions of annotated snRNAseq-resolved cell types to bulk RNA-seq WGCNA modules. **(A)** Uniform manifold approximation and projection (UMAP) plot showing major liver cell types. **(B)** Dot plot displaying expressions of marker genes in each cell type. Color indicates the average expression. Circle size indicates the percentages of cells that express the gene. **(C)** UMAP plot showing cells split by fibrosis stage. **(D)** Percentages of each cell type across individual MASH patients. On the x-axis, patient IDs are color-coded: blue indicates MASH patients with mild fibrosis, and red indicates those with advanced fibrosis. **(E)** Percentages of each cell type between mild fibrosis and advanced fibrosis. **(F)** Heatmap showing pearson correlation of deconvoluted cell fractions (columns) and module eigengenes (rows). Only correlations with *P* < 0.05 are shown. Larger dot represents higher absolute correlation coefficients. **(G)** Scatter plots showing MASH samples plotted by cell fractions of select liver cell types on the y-axis and the M3, M8, M1 and M6 module eigengene on the x-axis.

### 3.6 Characterization of HSCs subpopulations and intercellular communication in MASH fibrosis

Several studies have highlighted the importance of HSCs in driving the progression of liver fibrosis in MASH patients. Central to MASH pathogenesis is the conversion of HSCs into activated HSCs (aHSCs), characterized by excessive production of ECM. After quality control, we obtained 1,747 HSCs from the snRNA-seq data. We first compared the distribution of HSCs between mild and advanced fibrosis and found no significant differences between the two groups (Figure 5A). HSCs from MASH with advanced fibrosis expressed an altered transcriptomic profile, with 89 significantly overexpressed and 90 significantly repressed genes compared with HSCs from mild fibrosis (Figure 5B; Supplementary Table S12). To study the functions of HSCs during fibrosis progression in MASH, we performed enrichment analysis. Gene enrichment analysis suggested that these genes were particularly enriched in pathways for receptor-mediated virion attachment to host cell (GAS6 and ICAM1), neuron projection regeneration (NREP and PTN), cellular glucose homeostasis (HKDC1, GAS6 and CYP7A1) and positive regulation of protein localization to membrane (RAMP3, MTCL1, ARHGEF16 and YWHAG) (Figure 5C). Previous mouse and human studies have indicated high heterogeneity among HSCs in MASH livers (Wang et al., 2023; Yashaswini et al., 2024). We subdivided the HSCs into five clusters, which we termed HSC_0 to HSC_4 (Figure 5D). The distribution of these HSCs subsets did not vary significantly between fibrosis stages (Figure 5E; Supplementary Figure S6A,B; Supplementary Table S13). To fully annotate the HSCs, we identified different subcluster marker genes (Figure 5F). Notably, HSC_1 displayed a gene expression pattern similar to that of activated HSCs, expressing markers of activation, such as COL1A1 and TIMP2 (Figure 5G), which represented MASH fibrosis-associated HSCs. The upregulated genes in HSC_1 were mainly linked to pathways that are known drivers of fibrosis, including extracellular structure organization, extracellular matrix organization, external encapsulating structure organization, collagen fiber organization, and the regulation of cell migration. (Figure 5H). Given that HSCs activation and liver fibrosis involve complex intercellular crosstalk (Ramachandran et al., 2019; Watson et al., 2025), we employed ligand-receptor analysis to infer cell-cell interactions in MASH livers using the CellPhoneDB software. Remarkably, based on the cell interaction network counts (Supplementary Table S14), HSCs showed the strongest interactions with cholangiocytes and hepatocytes compared with all other HSC-interacting cell types (Figure 5I), supporting the notion that these cell populations play a central role in molding the HSCs phenotype and fostering the fibrotic niche. In particular, crosstalk between HSCs and hepatocytes has been widely implicated in fibrosis progression (Wang et al., 2016; Filliol et al., 2022; Sugimoto et al., 2025). In fibrotic livers, hepatocytes are frequently observed in proximity to aHSCs within scarred regions. However, intercellular communication between HSCs and cholangiocytes remains poorly understood. Thus, further investigation is required to delineate the roles of cholangiocytes and hepatocytes in MASH fibrosis at a single-cell resolution.

**FIGURE 5:**
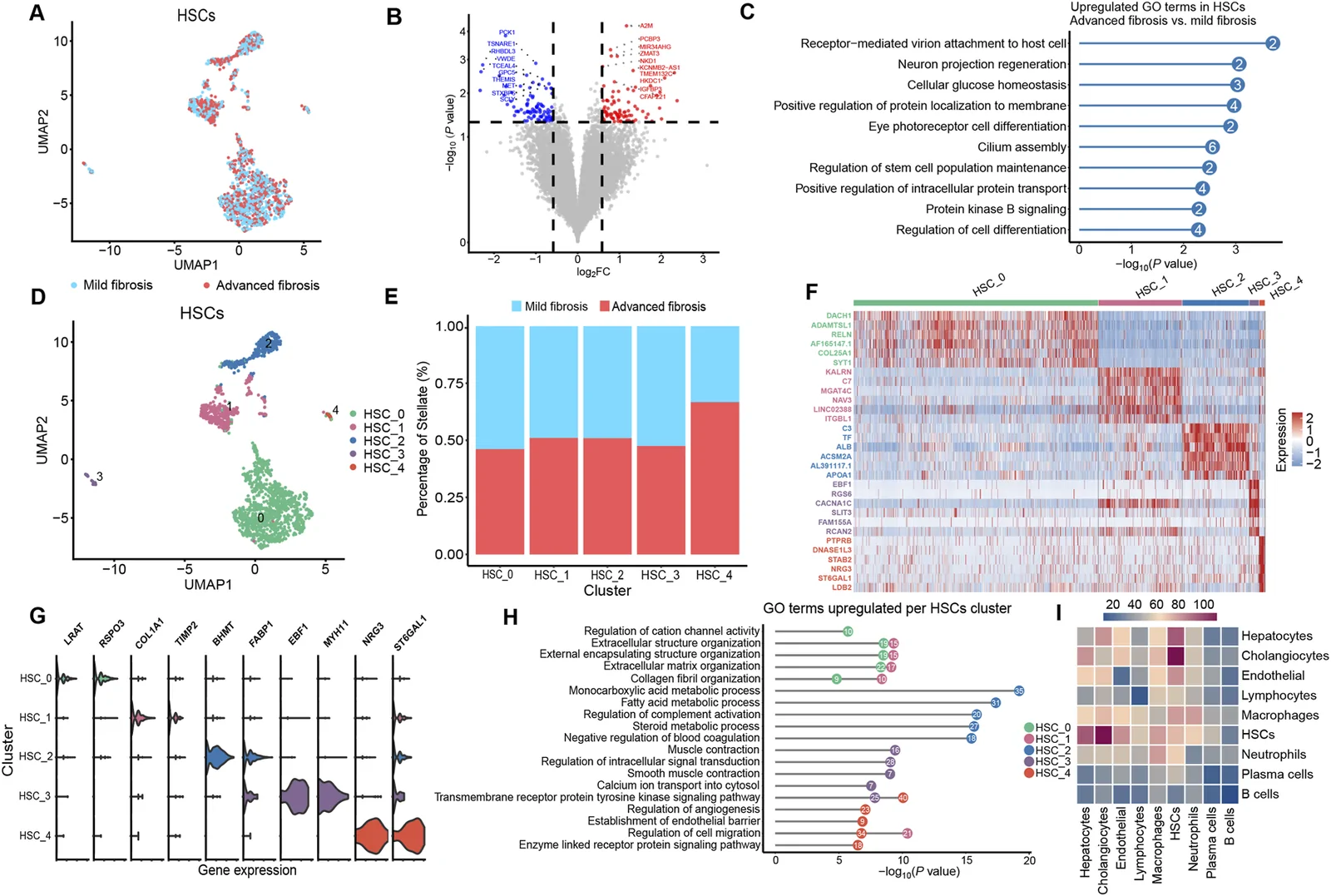
Characterization of hepatic stellate cells (HSCs) subsets in MASH livers with fibrosis. **(A)** UMAP plot showing HSCs split by fibrosis stage. **(B)** Volcano plot showing DEGs in HSCs comparing MASH with advanced fibrosis to those with mild fibrosis. **(C)** Lollipop plots displaying the top ten GO terms of biological processes enriched by upregulated DEGs identified in HSCs (advanced fibrosis vs. mild fibrosis). **(D)** UMAP plot showing HSC subclusters. **(E)** Percentages of HSC subsets between mild fibrosis and advanced fibrosis. **(F)** Heatmap showing the overexpressed genes between each subcluster and all other HSC subclusters. **(G)** Violin plots depicting expression levels of HSC cluster makers across HSC subclusters. **(H)** Lollipop plots showing the top five GO terms of biological processes enriched by upregulated DEGs identified in each HSC subclusters. **(I)** Heatmap showing the cell-cell interactions between major cell types. Color indicates the number of ligand-receptor pairs.

### 3.7 Identifying cholangiocyte subpopulations and key transcription factors driving fibrosis in MASH

Next, we explored the snRNA-seq gene signatures underlying the heterogeneity of cholangiocytes (CCs) in MASH livers. We observed 2,390 high-quality CCs in the liver from all patients with MASH, providing a transcriptomic atlas of tissue CCs (Figure 6A). The DEG analysis of all CCs revealed 103 upregulated genes in MASH with advanced fibrosis (Figure 6B; Supplementary Table S15). Gene enrichment analysis suggested that these genes were particularly enriched in pathways for regulation of lipoprotein lipase activity (ANGPTL8, APOC1 and PCSK6), induction of bacterial agglutination (FGB and FGA), regulation of vasoconstriction (FGB, FGA and KCNMB4), carbohydrate phosphorylation (TKFC and HK1) and positive regulation of peptide secretion (FGB and FGA) (Figure 6C). Unsupervised clustering of all CCs revealed their heterogeneity and identified four transcriptomically distinct cell subclusters, named CC_0 through CC_3 (Figure 6D). We then computed the fraction of cells in each CC subcluster to detect changes in the relative fractions (Figure 6E), and the distribution of CC subsets did not differ significantly across fibrosis stages. (Supplementary Figure S6C,D; Supplementary Table S13). Interestingly, CC_1 was characterized by the specific expression of FGF13 and PPARGC1A (Figures 6F,G), indicating a distinct subpopulation of CCs in the human liver and a potential target for MASH fibrosis. Additionally, CC_1 showed low ambient RNA contamination and doublet rates (Supplementary Figure S7A,B), supporting the robustness of this subcluster. Therefore, DecontX-corrected counts and doublet-filtered nuclei were not used in the final clustering. GO enrichment analysis showed that upregulated genes in CC_1 were primarily associated with pathways such as regulation of the Wnt signaling pathway, synapse organization, synapse assembly, regulation of signal transduction, and intrinsic apoptotic signaling in response to endoplasmic reticulum stress (Figure 6H).

**FIGURE 6:**
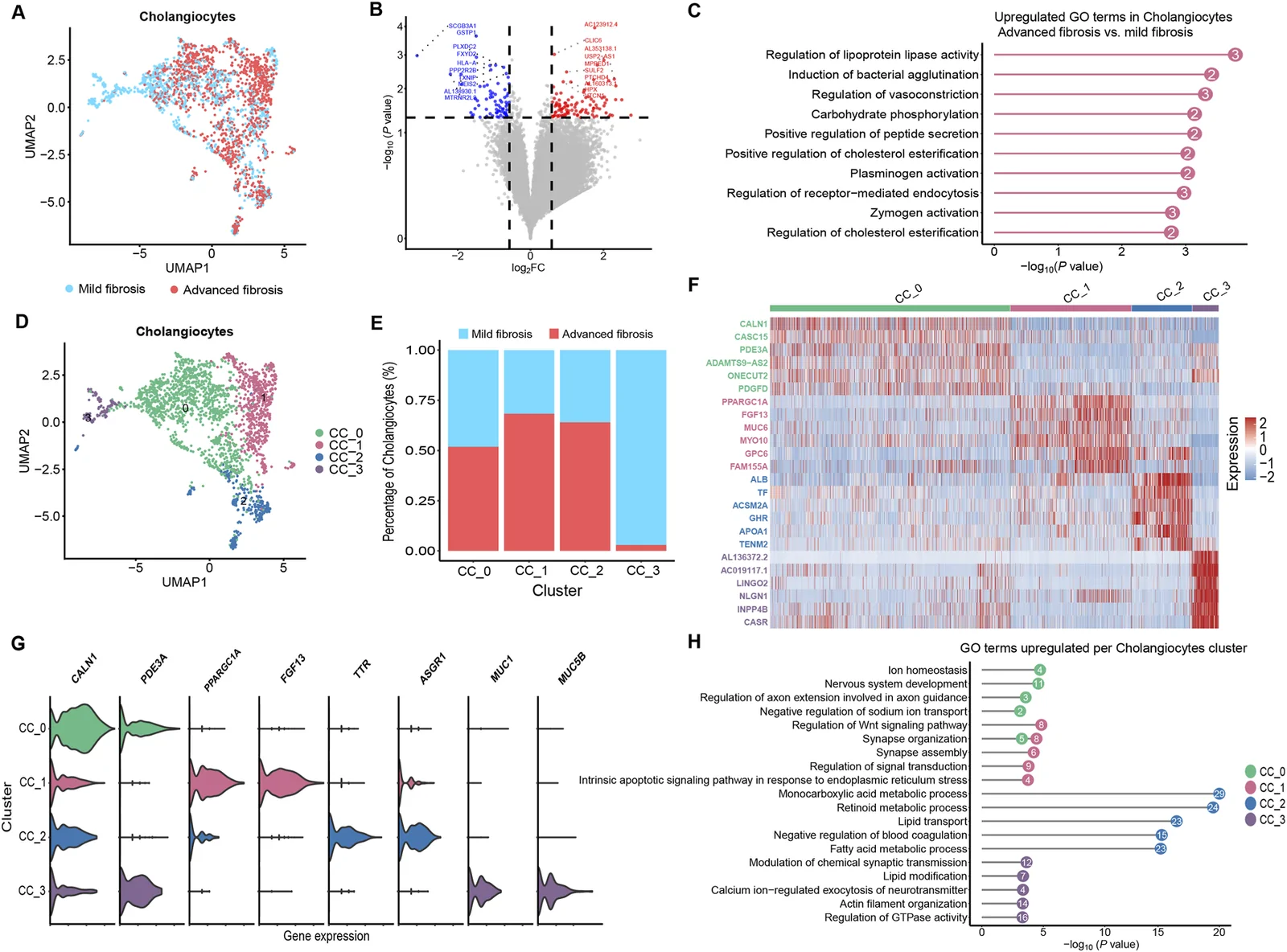
Characterization of cholangiocytes subsets in MASH livers with fibrosis. **(A)** UMAP plot showing cholangiocytes split by fibrosis stage. **(B)** Volcano plot showing DEGs in cholangiocytes comparing MASH with advanced fibrosis to those with mild fibrosis. **(C)** Lollipop plots displaying the top ten GO terms of biological processes enriched by upregulated DEGs identified in cholangiocytes (advanced fibrosis vs. mild fibrosis). **(D)** UMAP plot depicting cholangiocyte subclusters. **(E)** Percentages of cholangiocyte subsets between mild fibrosis and advanced fibrosis. **(F)** Heatmap showing the overexpressed genes between each subcluster and all other cholangiocyte subclusters. **(G)** Violin plots showing expression levels of cholangiocyte cluster makers across cholangiocyte subclusters. **(H)** Lollipop plots showing the top five GO terms of biological processes enriched by upregulated DEGs identified in each cholangiocyte subclusters.

Furthermore, by leveraging CellPhoneDB (Efremova et al., 2020) to analyze ligand–receptor interactions, we observed that among all CC subpopulations interacting with HSCs, CC_1 exhibited the strongest interactions (Figure 7A; Supplementary Table S16). Specifically, we focused this analysis only on receptor-ligand interactions that are uniquely enriched between CC_1 and HSCs. For instance, in HSCs, VEGFA can interact with NRP1 expressed in CC_1 (Figure 7B), while CC_1 expressed VEGFB and APP, respectively, to signal to HSCs through NRP1 and CD74 (Figure 7C). In addition, growth factor-related interactions, such as FGFR2–XPR1 and FGFR1–FGFR2, were more prominent between CC_1 and HSCs (Figure 7B). We then sought to examine the regulatory network underlying the CC_1 subpopulation and identified several activated regulons, highlighting CREB5, which ranked among the top ten regulons of CC_1 (Figure 7D). CREB5 displayed high regulatory activity and gene expression, specifically in the CC_1 subset (Figure 7E). GO enrichment analyses indicated that CREB5 target genes were significantly enriched in immune-related processes, including lymphocyte differentiation, the MAPK cascade, T cell receptor signaling, and B cell differentiation (Figure 7F). Pathway analysis using the hallmark gene set from human MSigDB revealed that the downstream targets of CREB5 also participated in immune- and inflammatory-related pathways, such as epithelial mesenchymal transition, IL-2/STAT5 signaling, TNF-alpha signaling via NF-κB, and IL-6/JAK/STAT3 signaling (Figure 7G). Accordingly, CREB5 expression was elevated in the livers of multiple MASH animal models compared with controls, and generally showed an increasing trend in human MASH (Figure 7H).

**FIGURE 7:**
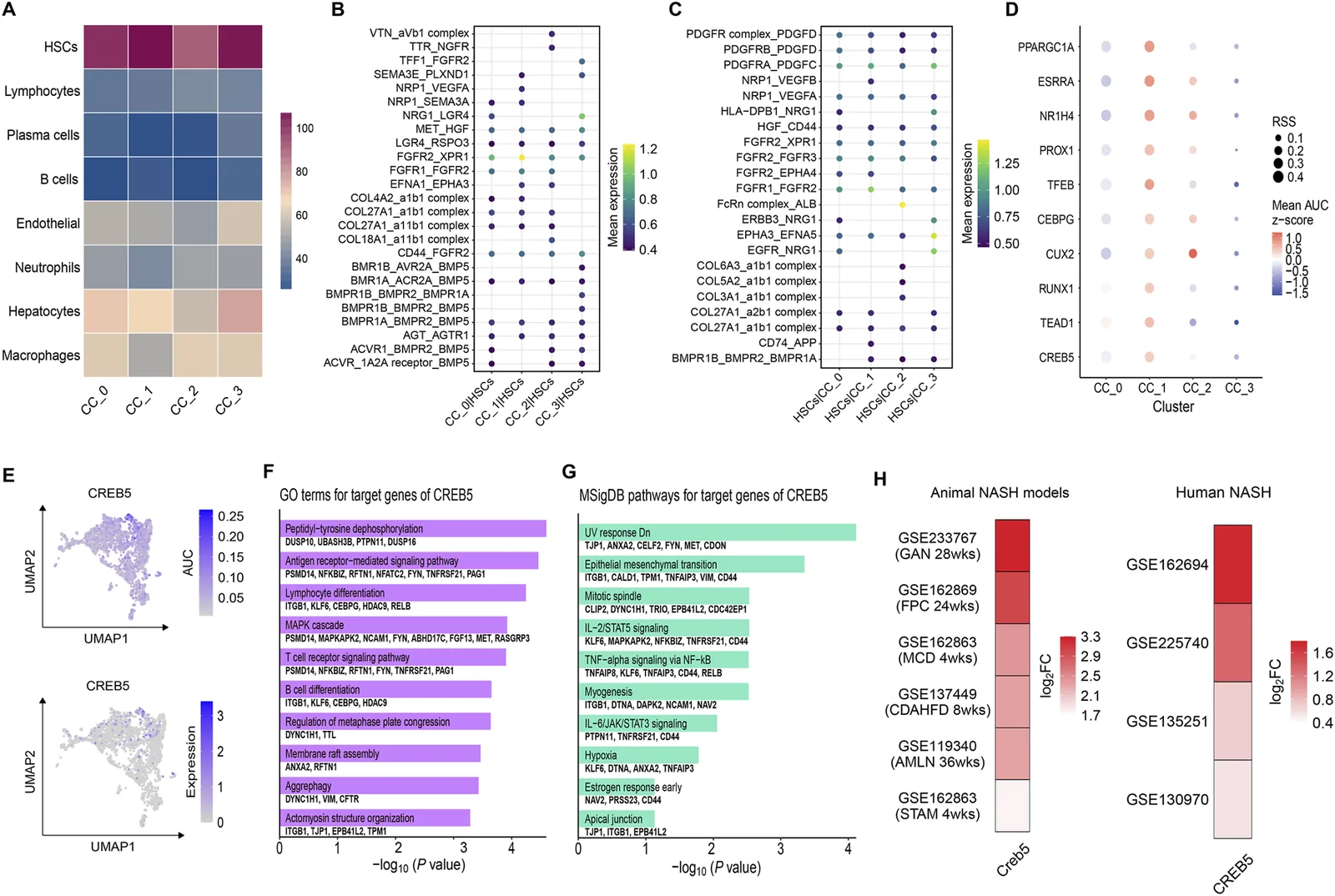
Identifying ligand-receptor interactions and transcription factor regulons of FGF13^+^ cholangiocytes in MASH livers with fibrosis. **(A)** Heatmap showing the cell-cell interactions between cholangiocyte subclusters and all other liver cells populations. Color corresponds to the number of ligand-receptor pairs. **(B, C)** Dot plots showing the significant ligand-receptor interactions between cholangiocyte subclusters and HSCs. **(D)** Dot plot displaying the top ten enriched regulons ranked by regulon specificity score (RSS) in FGF13^+^ cholangiocytes (CC_1). Colors represent scaled mean AUC scores for regulons across subclusters. Sizes represent fraction of cells in subcluster with regulon activity. **(E)** UMAP plot demonstrating the activity (up) and expression (down) of CREB5 in cholangiocyte subclusters. **(F)** Bar plot showing the top ten GO terms of biological processes enriched by target genes of CREB5. **(G)** Bar plot showing the top ten hallmark pathways enriched by target genes of CREB5. **(H)** Heatmap showing the log_2_FC in CREB5 expression (RNA-seq) in various mouse models of MASH (left) and in indicated human cohorts of MASH with fibrosis (right).

### 3.8 Identification of a hepatocyte subpopulation with increased SAAs expression in MASH with advanced fibrosis

Next, we analyzed hepatocytes (HEPs), the predominant cell population in this transcriptomic atlas (17,956 and 17,837 liver cells from MASH with mild fibrosis and advanced fibrosis, respectively) (Figure 8A). DEG analysis of HEPs between advanced fibrosis and mild fibrosis samples identified the upregulation of MCTP2, KCNAB2, PLUT1, GUCY1A1 and others in advanced fibrosis samples (Figure 8B; Supplementary Table S17). GO enrichment analysis revealed that the upregulated genes in HEPs from MASH with advanced fibrosis were enriched in camera-type eye photoreceptor cell differentiation (SOX9 and PROM1), glucose transmembrane transport (SLC2A14, SORT1 and SLC2A5), regulation of glial cell differentiation (EPHA4 and TNFRSF21), and positive regulation of chondrocyte differentiation (SOX9 and GDF5) (Figure 8C). Unsupervised clustering of HEPs delineated five distinct subsets (HEP_0 to HEP_4) (Figure 8D). We examined the distribution of HEP subsets across fibrosis stages and found no significant differences between stages. (Figure 8E; Supplementary Figure S6E,F; Supplementary Table S13). DEG analysis revealed that HEP_2 cells had high expression of SAA1 and SAA2 (Figures 8F,G). In addition, HEP_2 exhibited low ambient RNA contamination and doublet rates (Supplementary Figure S7C,D), supporting the robustness of this subclusters. Accordingly, the final clustering was conducted without DecontX correction or doublet filtering. Upregulated genes in the SAAs^+^ HEP subtype were primarily enriched in immune- and lipid metabolism-related biological processes, including regulation of complement activation, regulation of immune effector process, regulation of humoral immune response, sterol biosynthetic process, and secondary alcohol biosynthetic processes (Figure 8H), revealing an innate immune-related response and hepatocyte injury in the HEP_2 subtype. As expected, we found that SAA1 and SAA2 were significantly upregulated in bulk transcriptomes from the liver tissues of several MASH mouse models and patients (Figure 8I).

**FIGURE 8:**
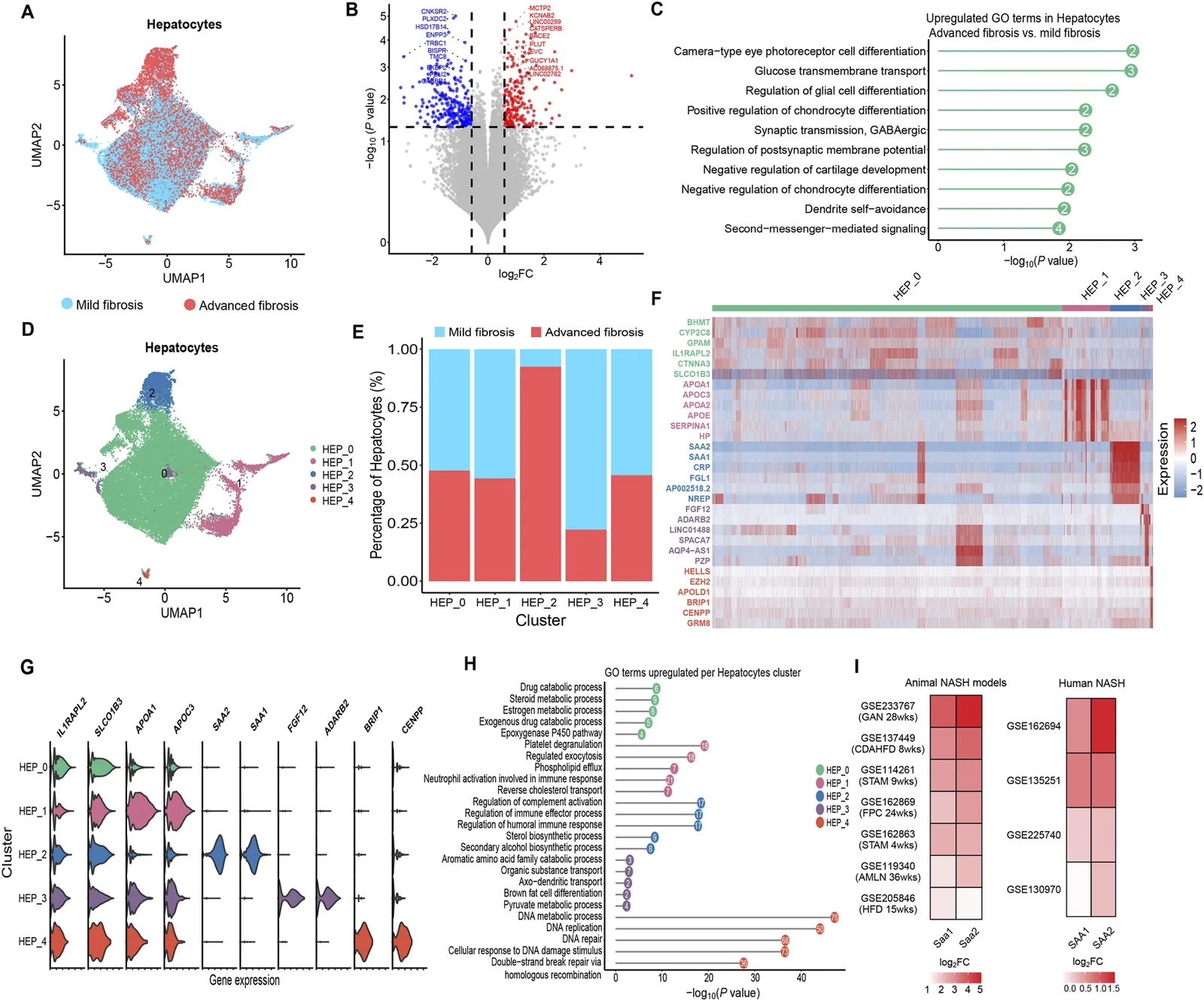
Characterization of hepatocytes in MASH livers with fibrosis. **(A)** UMAP plot showing hepatocytes split by fibrosis stage. **(B)** Volcano plot showing DEGs in hepatocytes comparing MASH with advanced fibrosis to those with mild fibrosis. **(C)** Lollipop plots exhibiting the top ten GO terms of biological processes enriched by upregulated DEGs identified in hepatocytes (advanced fibrosis vs. mild fibrosis). **(D)** UMAP plot showing hepatocyte subclusters. **(E)** Percentages of hepatocyte subsets between mild fibrosis and advanced fibrosis. **(F)** Heatmap demonstrating the overexpressed genes between each subcluster and all other hepatocyte subclusters. **(G)** Violin plots showing expression levels of hepatocyte cluster makers across hepatocyte subclusters. **(H)** Lollipop plots showing the top five GO terms of biological processes enriched by upregulated DEGs identified in each hepatocyte subclusters. **(I)** Heatmap showing the log_2_FC in SAA1 and SAA2 expression (RNA-seq) in various mouse models of MASH (left) and in indicated human cohorts of MASH with fibrosis (right).

### 3.9 Characterization of transcription factors and ligand–receptor interactions of SAAs^+^ hepatocyte subpopulation

To further uncover the novel regulator of SAAs^+^ hepatocytes, we performed SCENIC analysis to evaluate the top specifically expressed transcription factors (TFs) ant their activities in the TF regulatory network. Notably, STAT3, a potent positive regulator correlated with liver fibrosis progression in patients with MASLD (Jiao et al., 2023), emerged among the top-ten regulons in HEP_2 compared to other HEP subsets based on the snRNA-seq data (Figure 9A). STAT3 exhibited high regulatory activity and gene expression levels in the regulatory network of SAAs^+^ hepatocytes (Figure 9B), and its regulatory activity showed the strongest correlation with the expression levels of the SAA1 and SAA2 genes (Figure 9C). GO enrichment analysis indicated that the target genes regulated by STAT3 were involved in complement- and immune-related biological processes, such as regulation of complement activation, regulation of immune effector processes, and regulation of humoral immune response (Figure 9D). MSigDB enrichment analysis further revealed that STAT3 target genes were enriched in pathways related to coagulation, TNF-α signaling via NF-kB, and IL-6/JAK/STAT3 signaling (Figure 9E). Together, these results suggest that the activation of STAT3 in hepatocytes and the subsequent production of SAAs triggered the intrahepatic inflammatory response and complement system that led to MASH fibrogenesis. SAA1 and SAA2 may be potential therapeutic targets for MASH fibrosis. Having seen an extensive transcriptional regulatory network of hepatocyte subpopulations, we next investigated the specific interactions between these subpopulations and other cell types in all MASH livers using CellPhoneDB. Within this ligand-receptor interaction analysis, we found strong cellular communication between the HEP_2 subpopulation (SAAs^+^ hepatocytes) and both HSCs and macrophages, which are the key mediators of liver inflammation and fibrogenesis (Figure 9F; Supplementary Table S18). HSCs could signal to HEP_2 cells through the expression of RSPO3, which can be recognized by the LGR4 receptor in HEP_2 cells (Figure 9G). The expression of IGF1 by HEP_2 is recognized by IGF1R, which is expressed by HSCs (Figure 9G). In addition, COL27A1_a11b1 and COL4A4_a1b1 complex interactions were observed between the HEP_2 cells and HSCs (Figures 9G,H). TNFSF14, expressed by HEP_2, was recognized by TNFRSF14 expressed by macrophages (Figure 9I). NAMPT from HEP_2 could also signal to macrophages expressing P2RY6, whereas HEP_2 expression of IGF1 could signal to IGF1R expressed on macrophages (Figure 9I). Furthermore, specific interactions, including SELE_GLG1, ICAM1_ITGAL, and CD44_SELE, were observed between HEP_2 cells and macrophages (Figures 9I,J).

**FIGURE 9:**
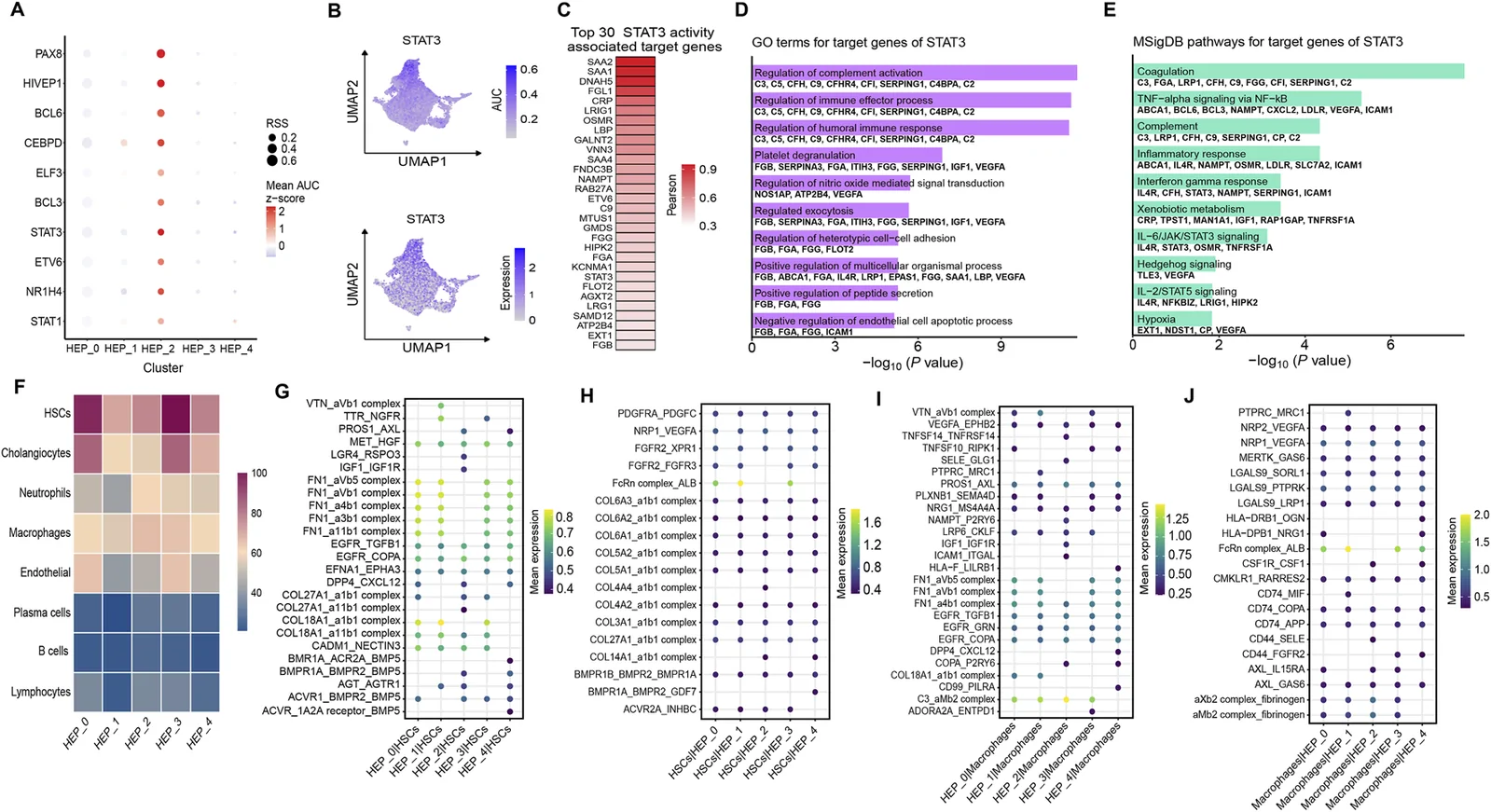
Identifying transcription factor regulons and ligand-receptor interactions of SAAs^+^ hepatocytes in MASH livers with fibrosis. **(A)** Dot plot showing the top ten enriched regulons ranked by regulon specificity score (RSS) in SAAs^+^ hepatocytes (HEP_2). Color indicates scaled mean AUC scores for regulons across subclusters. Size represents fraction of cell in subcluster with regulon activity. **(B)** UMAP plot showing the activity (up) and expression (down) of STAT3 in hepatocyte subclusters. **(C)** Heatmap showing the correlation between STAT3 regulatory activity and the expression levels of its top 30 target genes. Color indicates the Pearson correlation coefficients. **(D)** Bar plot exhibiting the top ten GO terms of biological processes enriched by target genes of STAT3. **(G)** Bar plot showing the top ten hallmark pathways enriched by target genes of STAT3. **(F)** Heatmap demonstrating the cell-cell interactions between hepatocyte subclusters and all other liver cells populations. Color indicates the number of ligand-receptor pairs. **(G, H)** Dot plots showing the significant ligand-receptor interactions between hepatocyte subclusters and HSCs. **(I, J)** Dot plots displaying the significant ligand-receptor interactions between hepatocyte subclusters and macrophages.

## 4 Discussion

The severity of liver fibrosis is closely linked to morbidity and mortality in patients with MASH and serves as a pivotal predictor of severe liver-related outcomes, including cirrhosis and HCC (Friedman et al., 2018; Huang et al., 2023). Although recent multi-omics studies have identified numerous molecules and cellular subpopulations associated with MASLD/MASH fibrosis, the underlying molecular mechanisms remain incompletely understood (Bai et al., 2023; Kim et al., 2025; Martin et al., 2025). In this study, our integrative analysis established a 7-gene transcriptomic signature capable of discriminating mild fibrosis from advanced fibrosis in MASH, while simultaneously identifying key cellular subpopulations, transcriptional regulators, and ligand-receptor interactions associated with fibrosis severity. Collectively, these findings provide novel mechanistic insights into the pathogenesis of MASH fibrosis and a roadmap for the development of potential targeted therapies.

To our knowledge, this is the first comprehensive bioinformatic analysis of hepatic bulk RNA-seq data to systematically explore key genes, gene network modules, and enriched molecular pathways involved in the progression of fibrosis among MASH patients. By leveraging network analysis and multiple machine learning algorithms, we uncovered a 7-gene signature (CAPN6, FAP, KRT23, SOX4, STMN2, TAGLN, and VWF) that may serve as a candidate set of biomarkers for liver fibrosis classification, with each gene playing a role in the regulation and progression of fibrosis. Notably, VWF is a glycoprotein that is canonically secreted by vascular endothelial cells (ECs) and is central to platelet adhesion, aggregation, and thrombosis. Of note, emerging evidence from RNAscope and 3D imaging approaches has illustrated that VWF^+^ scar-associated ECs penetrate fibrotic septa in murine MASH, engaging activated HSCs and exhibiting *in situ* protease activity, thus functioning as active regulators of fibrosis regression (Li et al., 2026). Additionally, analyses of published gene expression datasets from MASLD/MASH patients revealed marked upregulation of STMN2 across advancing fibrosis stages, considering it as a hub gene significantly correlated with fibrosis severity (Hu and Zhou, 2023). TAGLN, a prototypical actin-binding protein localized to stress fibers and other cytoskeletal structures, regulates cell migration and morphological remodeling (Yan et al., 2025). A clinical study evaluating serum biomarkers in MASLD patients demonstrated that serum TAGLN concentrations correlated with MASLD severity and appeared to track hepatic fibrosis progression, supporting its potential as a non-invasive fibrosis biomarker (Zwolak et al., 2016). Beyond the aforementioned well-characterized fibrosis-related genes, CAPN6 belongs to the non-classical calpain family and plays important roles in microtubule stabilization, cell migration, and apoptosis regulation (Chen et al., 2020). Direct investigations of CAPN6 in MASH fibrosis remain limited, however, its aberrant expression has been documented across a broad range of diseases including cancer, cardiovascular disease, neurological disorders, and metabolic diseases, suggesting a potential role in cross-disease pathophysiological regulation (Chen et al., 2020). In summary, the 7-gene signature identified in this study not only deepens our mechanistic understanding of MASH-related fibrosis at the molecular level, but also provides a tissue signature associated with advanced fibrosis. This signature could help resolve issues in translational research, such as cross-cohort reproducibility and patient stratification, and generate hypotheses about mechanisms driving MASH fibrosis progression.

Bulk transcriptome analyses have identified gene signatures associated with MASH fibrosis; however, the cellular heterogeneity of the liver obscures the cell-type origins of these signals. To address this, we performed deconvolution analysis integrating bulk-based coexpression networks with snRNA-seq data and linking distinct gene networks to their putative cellular sources in advanced MASH fibrosis, which provides a foundation for subsequent single-cell resolution investigations of the molecular mechanisms underlying MASH fibrosis.

In MASH, HSCs represent the principal cell type implicated in ECM production and play a central pathogenetic role in the progression of liver fibrosis (Wang et al., 2023; Bendixen et al., 2024). Herein, we identified five HSC subclusters, among which COL1A1^+^ and TIMP2^+^ cells represented the highly fibrogenic aHSCs subpopulation. COL1A1, which is co-expressed with COL1A2 and COL3A1, defines the hallmark fibrogenic transcriptional signature of aHSCs and has been consistently proposed as a key marker for aHSCs in previous studies (Rosenthal et al., 2021; Yang et al., 2021; Kim et al., 2025). TIMP2, another defining marker of this subpopulation, is broadly recognized as a profibrotic mediator. Mechanistically, TIMP2 suppresses metalloproteinase activity to impair ECM degradation and promote fibrotic tissue accumulation in HSCs (Nie et al., 2010; Xue et al., 2021). Beyond HSC intrinsic heterogeneity, interactions between HSCs and other hepatic cell types play crucial roles in MASH pathogenesis. Our ligand-receptor interaction network analysis revealed robust and significantly enriched cell-cell communication between cholangiocytes, hepatic parenchymal cells, and HSCs, corroborating the established importance of these intercellular interactions in MASH-driven fibrogenesis.

As a next step, our snRNA-seq analysis identified a distinct cholangiocyte subset (CC_1) characterized by the specific expression of FGF13 and PPARGC1A. Although the function of FGF13 in the liver remains unclear, available evidence points to its potential roles in regulating cholangiocyte transdifferentiation into hepatocytes (Gribben et al., 2024; Hammond et al., 2025). A recent study identified FGF13 as a critical ceramide-dependent mediator of energy and glucose homeostasis, highlighting its potential as a therapeutic target for obesity-associated metabolic dysfunction, including insulin resistance and hepatic steatosis (Naderi et al., 2025). Hepatic loss of PPARGC1A has also been shown to drive fibrosis independently from lipid and glucose metabolism in mouse model of MASH (Piccinin et al., 2023). Moreover, our cell-cell interaction analysis revealed specific receptor-ligand pairs between CC_1 and HSCs. HSC-derived VEGFA interacts with the NRP1 receptor on CC_1 and CC_1-derived VEGFB interacts with NRP1 on HSCs. Notably, the VEGFA and NRP1 ligand-receptor pair was previously observed by Hess et al. suggesting a critical role of VEGFA-mediated HSC-cholangiocyte interaction in the development of hepatic fibrosis (Hess et al., 2023). Mechanistically, previous reports in mice revealed that VEGFA significantly induces biliary epithelial cell-to-hepatocyte conversion, consequently alleviating liver steatosis and fibrosis (Rizvi et al., 2023). We also observed less studies on the interactions between HSCs and CC_1, including the CD74_APP, FGFR2_XRP1, and FGFR1_FGFR2 signaling axes. Notably, the APP_CD74 ligand-receptor axis has been implicated as a critical regulatory mechanism in the pathogenesis of atherosclerosis, glioblastoma multiforme (GBM), and kidney fibrosis (Liu et al., 2024; Ma et al., 2024; Zhang K. et al., 2025). Furthermore, we identified CREB5 (cAMP-response element binding protein 5) as a key transcriptional regulator specifically active in the CC_1 cholangiocyte subset, with its target genes predominantly enriched in immune-related signaling pathways and epithelial-mesenchymal transformation (EMT). Consistent with our observations, a recent study demonstrated that overexpression of CREB5 promotes EMT in HCC cells (Wang et al., 2025), suggesting that CREB5 may enhance cholangiocyte EMT via inflammatory pathways (e.g., NF-κB) in MASH-associated fibrosis. In the context of fibrotic disease, CREB5 has further been shown to activate fibronectin (Fn1) expression in renal fibrosis, thereby facilitating ECM deposition (Su et al., 2021). In support, we discovered that CREB5 expression was consistently elevated across multiple MASH models and human datasets, suggesting a functional role in MASH pathogenesis rather than a merely reactive response. Despite these converging lines of evidence, the specific contribution of CREB5 to liver fibrosis, particularly in MASH, remains largely unexplored. Collectively, our findings suggest that CREB5 is a promising candidate regulator and potential therapeutic target for MASH-related fibrosis.

Our study further revealed the heterogeneity of hepatocytes in MASH fibrosis progression. We observed a marked increase in SAAs^+^ hepatocyte (HEP_2) frequency in MASH with advanced fibrosis versus mild fibrosis, with upregulated genes in HEP_2 predominantly enriched in immune response and lipid metabolism pathways. SAA family proteins, particularly SAA1 and SAA2, are well-established acute-phase reactants in inflammatory responses, primarily expressed and secreted by hepatocytes and adipocytes (Jalkanen et al., 2026). Elevated hepatic SAA1 expression has been reported in fatty liver from patients with MASLD and murine models (Jiang et al., 2022), which is consistent with our current observations. Mechanistically, SAA1 triggers hepatic lipid accumulation and inflammatory activation through a SAA1/TLR4/NF-κB feedforward circuit (Jiang et al., 2022), while SURF4-mediated hepatic SAA1 secretion activates HSCs to drive fibrogenesis (Wu et al., 2023). Beyond the liver, elevated SAA proteins are linked to obesity, T2DM, hypertension, and heart failure with preserved ejection fraction (HFpEF) (Liu et al., 2026). Strikingly, SAA1 and SAA4 were recently identified as overlapping plasma proteomic features in both MASLD and HFpEF, associated with increased transcription in the heart of genes involved in ECM remodeling (Strocchi et al., 2024). Together, these findings position SAAs^+^ hepatocytes as a functional subpopulation and a potential therapeutic target for MASH-associated fibrosis.

Furthermore, transcriptional regulatory network analysis identified STAT3 as a key regulator of SAA1/2 expression in the HEP_2 subpopulation, a well-established role in multiple studies (Lee et al., 2019; Wu et al., 2023; Stone et al., 2024; Jalkanen et al., 2026). In liver cancer, Wu et al. demonstrated that IL-6 secreted by tumor cells could induce activation of JAK-STAT3 signaling in hepatocytes and the subsequent production of SAA by hepatocytes, which could further promote local immunosuppression and tumor progression. Intriguingly, our study revealed that STAT3 target genes in SAAs^+^ hepatocytes were not exclusively enriched in the IL-6/JAK/STAT3 pathway, but also converged on coagulation, inflammatory response, and TNF-α signaling via NF-κB, implicating additional pathways that may be altered in SAAs^+^ hepatocytes in advanced MASH fibrosis. Cell-cell communication analysis further showed that HEP_2 engages in prominent interactions with HSCs; specifically, HSCs and HEP_2 interact via the RSPO3_LGR4 and IGF1_IGF1R axes, with additional interactions involving COL27A1-a11b1 and COL4A4-a1b1 complexes. The RSPO3_LGR4 interaction, previously identified as a high-frequency ligand-receptor interaction pair between HSCs and hepatocytes in chronic liver disease (CLD) and mouse liver (Sugimoto et al., 2025), promotes Wnt/β-catenin signaling to regulate hepatocyte differentiation and metabolic function. Notably, RSPO3 mRNA expression in HSCs was progressively downregulated with advancing fibrosis during MASLD. Thus, RSPO3-LGR4-mediated HSC-hepatocyte crosstalk appears to play a role in liver fibrogenesis, presenting a promising therapeutic target. The IGF1_IGF1R ligand-receptor pair also exerts an influence on MASH-associated fibrosis. Hepatocytes represent the principal source of IGF1 in circulation, while IGF1R is abundantly expressed on HSCs, both of which play pivotal roles in the progression of liver fibrosis. Nishizawa et al. showed that IGF1 can ameliorate steatosis, inflammation, and fibrosis in mouse models of MASH and cirrhosis (Nishizawa et al., 2016).

Similarly, previous research has highlighted that signal exchange between hepatocytes and macrophages is particularly prominent in MASLD/MASH. A recent study integrating snRNA-seq with spatial transcriptomics revealed that periportal hepatocytes secrete SAA1 to recruit macrophages, which subsequently engage CADM1-mediated intercellular signaling to promote MASLD development (Zhong et al., 2026). In this study, we also identified robust intercellular interactions between HEP_2 and macrophages, mediated through the following ligand-receptor pairs: TNFSF14_TNFRSF14, SELE_CD44 and NAMPT_P2RY6. TNFSF14 and its receptor TNFRSF14 have been well-documented to play crucial roles in immunomodulation and inflammatory regulation across a spectrum of diseases, including MASH, MASH-related fibrosis, and steatotic liver disease-associated hepatocellular carcinoma (SLD-HCC) (Otterdal et al., 2015; Wang et al., 2021; Prawira et al., 2026). It has been recently reported that CD44 plays a critical role in the development of MASH by regulating hepatic macrophage polarization and infiltration (Patouraux et al., 2017). Moreover, SELE expression is strongly upregulated in the context of hepatic fibrosis and MASH, as consistently demonstrated in human studies (Simons et al., 2020; Lai et al., 2024). Taken together, these findings suggest that activation of the CD44 signaling pathway through SELE_CD44 ligand-receptor interaction may contribute to the progression of MASH-associated fibrosis. With respect to the NAMPT_P2RY6 axis, prior evidence indicates that NAMPT deficiency or functional impairment leads to reduced NAD^+^ levels, thereby exacerbating lipid metabolic dysregulation and inflammatory responses, which collectively accelerate MASLD progression (Zhang C.-Y. et al., 2025). Regarding P2RY6 (also known as P2Y6), genetic knockout of the purinergic P2Y6 receptor has been shown to be insufficient to ameliorate liver injury and inflammation in MASH, suggesting a context-dependent or compensatory role of this receptor in disease pathogenesis (Nishiyama et al., 2023).

This study has several limitations. First, the impact of batch effects on the results is a notable concern for all data-integration studies. Although batch correction strategies were applied, residual batch effects arising from heterogeneous liver tissue sampling procedures and sequencing protocols across multiple cohorts may introduce batch effects. Moreover, testing on ComBat-adjusted values with the group protected can overstate significance. Second, the current analysis relies solely on transcriptomics, without the integration of multi-omics data, thereby limiting the comprehensiveness of biological interpretation. Third, using a single-nucleus reference to deconvolve whole-tissue bulk RNA-seq data introduces a nuclear/cytoplasmic transcriptome mismatch, because snRNA-seq captures only nuclear RNA whereas bulk data contain both nuclear and cytoplasmic transcripts. Although CIBERSORTx S-mode correction, which directly adjusts the signature matrix for cross-platform batch effects, mitigated this bias, we acknowledge that residual bias may persist. Fourth, snRNA-seq inherently lacks a spatial context; future incorporation of spatial transcriptomics (ST) would enable simultaneous profiling of gene expression and cell-cell interactions within individual tissue sections, providing a more spatially resolved understanding of the cellular and molecular mechanisms involved in MASH fibrosis pathogenesis. Lastly, we acknowledge that functional experiments *in vivo* or *in vitro* are essential to validate our findings obtained from bioinformatic analyses, which is the primary limitation of this work and may see progress in the future. In addition, although we examined the expression of key genes in the MCD model, only male mice were used, and future studies including female mice are warranted.

## 5 Conclusion

Overall, we performed a comprehensive analysis of bulk and single-nucleus RNA-seq data of MASH-associated fibrosis using a systems biology approach. The results of this study will help provide candidate mechanisms and therapeutic targets for liver fibrosis in patients with MASH.

## Data Availability

Publicly available datasets were analyzed in this study. This data can be found here: The datasets analyzed for this study can be found in the Gene Expression Omnibus (GEO): GSE130970, GSE135251, GSE162694, GSE225740, GSE174478, GSE202379, GSE205846, GSE137449, GSE114261, GSE162863, GSE233767, GSE162869, and GSE119340.
