# Supplementary material for: Integrative machine learning analysis of bulk and single-nucleus RNA sequencing reveals key cell types and biomarkers driving MASH-associated fibrosis
Source: Front Cell Dev Biol. 2026 Oct 29;14:1961567. doi: 10.3389/fcell.2026.1961567 (PMC13754731; doi:10.3389/fcell.2026.1961567)
Supplement: Supplementary file 1 [file Supplementaryfile1.docx]

*Supplemental Materials*

**Combination of machine learning-based bulk and single-nucleus RNA sequencing reveals key cell types and biomarkers in MASH-associated fibrosis**

Junjie Zhang^1,2,3*^, Junjie Wang^1,2,3^, Yan Shen^4^, Yirong Zhang^1,2,3^, Zimeng Luo^1,2,3^, Xinyao Kong^1,2,3^, Wang Li^3,5^, Juan Luo^2,3^, Fei Xu^6*^ and Shaogui Wan^3,7*^

^1^Key Laboratory of Prevention and Treatment of Cardiovascular and Cerebrovascular

Diseases (Ministry of Education), Gannan Medical University, Ganzhou 341000, China.

^2^School of Basic Medicine, Gannan Medical University, Ganzhou 341000, China.

^3^Institute of Genomics and Precision Medicine, Gannan Medical University, Ganzhou

341000, China.

^4^School of Public Health and Health Management, Gannan Medical University, Ganzhou, 341000, China.

^5^School of Medical and Information Engineering, Gannan Medical University, Ganzhou, 341000, China.

^6^Department of Hepatology, The Affiliated Fifth People’s Hospital of Ganzhou, Gannan

Medical University, Ganzhou 341000, China.

^7^School of Medical Technology, Gannan Medical University, Ganzhou 341000, China.

^*^Correspondence to: J. Zhang, Key Laboratory of Prevention and Treatment of Cardiovascular and Cerebrovascular Diseases (Ministry of Education), Gannan Medical University, Ganzhou 341000, China; School of Basic Medicine, Gannan Medical University, Ganzhou 341000, China; Institute of Genomics and Precision Medicine, Gannan Medical University, Ganzhou 341000, China.

*E-mail addresses:* [zhangjunjielab@hotmail.com](mailto:zhangjunjielab@hotmail.com) (J. Zhang), [xufei8586@163.com](mailto:xufei8586@163.com) (F. Xu), [wansg@gmu.edu.cn](mailto:wansg@gmu.edu.cn) (S. Wan)

1 **Supplementary Figures and Tables**
  1.1 **Supplementary Figure Legends**

**Supplementary Figure S1** Batch correction of bulk RNA-seq data. **(A)** Principal component analysis (PCA) plot with batch effect uncorrected. **(B)** PCA plot with batch effect corrected using ComBat algorithm. (**C**) Principal component plot of samples based on top 500 most variable gene expression from combining gene expression profiles (MergeCohort). MASH with advanced fibrosis are marked in red; MASH with mild fibrosis are marked in blue. (**D**) Volcano plot displaying DEGs comparing MASH with advanced fibrosis to those with mild fibrosis in MergeCohort. DEGs are listed in Supplemental Table S6. (**E,F**) Lollipop plots showing the top ten GO terms of biological processes enriched by upregulated (**E**) and downregulated (**F**) DEGs identified in MASH with advanced fibrosis of MergeCohort.

**Supplementary Figure S2** Shared differentially expressed genes (DEGs) between the MergeCohort and each individual cohort. **(A-D)** Venn diagrams showing the overlap of DEGs between the MergeCohort and four cohorts: GSE130970 **(A)**, GSE135251 **(B)**, GSE162694 **(C)**, and GSE225740 **(D)**.

**Supplementary Figure S3** Comparison of the log_2_FC of DEGs between the MergeCohort and each individual cohort. **(A-D)** Scatter plots comparing the log_2_fold change (log_2_FC) of the shared DEGs between the MergeCohort (x axis) and individual cohort (y axis): GSE130970 **(E)**, GSE135251 **(F)**, GSE162694 **(G)**, and GSE225740 **(H)**. Pearson correlation coefficients (r) and corresponding p values are shown in the top-left corner of each plot. Selected representative genes are labeled.

**Supplementary Figure S4** Selection of soft-thresholding power for coexpression network analysis in MergeCohort. The left panel showing the scale-free fit index versus soft-thresholding power. The right panel representing the mean connectivity versus soft-thresholding power. Power 10 was selected, for which the fit index curve flattens out upon reaching a high value (> 0.85).

**Supplementary Figure S5 Heatmaps of 1og_2_FC for seven selected genes across multiple datasets with and without sex adjustment.** **(A)** Heatmap showing the 1og_2_FC of seven genes (CAPN6, FAP, KRT23, SOX4, STMN2, TAGLN, and VWF) in four independent datasets using the unadjusted model. **(B)** Heatmap showing the log2FC of the same seven genes in four independent datasets using the sex-adjusted model. The color scale represents the log_2_FC values. The numerical values within each cell indicate the specific log_2_FC and adjusted p-value for that gene in the corresponding dataset.

**Supplementary Figure S6** Comparative analysis of cell subtype composition across patients stratified by fibrosis stage. **(A-C)** Stacked bar plots showing the relative proportions of cell subtypes per patient in HSCs **(A)**, cholangiocytes **(B)**, and hepatocytes **(C)** subcluster. X axis labels are color-coded by fibrosis stage: blue indicates MASH patients with mild fibrosis, and red indicates MASH patients with advanced fibrosis. **(D-F)** Box plots comparing the percentage of each cell subtype between MASH patients with mild fibrosis (blue) and advanced fibrosis (red) for HSCs **(D)**, cholangiocytes **(E)**, and hepatocytes **(F)** subtypes. Benjamini–Hochberg (BH)-adjusted p values from two-sided Wilcoxon rank-sum tests are shown (*n* = 16 advanced fibrosis and *n* = 15 mild fibrosis).

**Supplementary Figure S7** Quality control metrics across hepatocytes and cholangiocytes cell subtypes. **(A,C)** Box plots showing the ambient mRNA contamination fraction (%) for hepatocytes subtypes and cholangiocytes subtypes, respectively. **(B,D)** Stacked bar plots showing the percentage of singlets (blue) and doublets (red) detected within hepatocytes subtypes and cholangiocytes subtypes, respectively.

**1.2 Supplementary Table Legends**

**Supplementary Table S1** The details of MASH datasets.

**Supplementary Table S2** DEGs identified in MergeCohort.

**Supplementary Table S3** GO terms of biological processes enriched by upregulated DEGs identified in MergeCohort.

**Supplementary Table S4** GO terms of biological processes enriched by downregulated DEGs identified in MergeCohort.

**Supplementary Table S5** Top 10 GO terms of biological processes enriched by module genes identified in MergeCohort

**Supplementary Table S6** GO terms of biological processes enriched by genes in the Brown and Red modules.

**Supplementary Table S7** DEGs in MASH with advanced fibrosis identified by Robust Rank Aggregation (RRA).

**Supplementary Table S8** Genes overlapped between RRA-identified DEGs, DEGs observed in MergeCohort and genes in the crucial WGCNA modules.

**Supplementary Table S9** KEGG pathways enriched by genes overlapped between RRA-identified DEGs, DEGs observed in MergeCohort and genes in the crucial WGCNA modules.

**Supplementary Table S10** The primer sequences.

**Supplementary Table S11** Canonical marker genes for cell type annotations in snRNA-seq data analysis.

**Supplementary Table S12** DEGs in hepatic HSCs cells (HSCs) comparing MASH with advanced fibrosis to those with mild fibrosis.

**Supplementary Table S13** Differential cell type abundance analysis by scCODA comparing mild fibrosis and advanced fibrosis.

**Supplementary Table S14** Cell-cell interaction count between major cell types.

**Supplementary Table S15** DEGs in cholangiocytes comparing MASH with advanced fibrosis to those with mild fibrosis.

**Supplementary Table S16** Cell-cell interactions between cholangiocyte subclusters and all other liver cells populations.

**Supplementary Table S17** DEGs in hepatocytes comparing MASH with advanced fibrosis to those with mild fibrosis.

**Supplementary Table S18** Cell-cell interactions between hepatocyte subclusters and all other liver cells populations.
